# 5-Nitroisoxazoles in *S_N_Ar* Reactions: A Novel Chemo- and Regioselective Approach to Isoxazole-Based Bivalent Ligands of AMPA Receptors

**DOI:** 10.3390/ijms242216135

**Published:** 2023-11-09

**Authors:** Dmitry A. Vasilenko, Nadezhda S. Temnyakova, Sevastian E. Dronov, Eugene V. Radchenko, Yuri K. Grishin, Alexey V. Gabrel’yan, Vladimir L. Zamoyski, Vladimir V. Grigoriev, Elena B. Averina, Vladimir A. Palyulin

**Affiliations:** 1 Department of Chemistry, Lomonosov Moscow State University, Leninskie Gory 1/3, 119991 Moscow, Russia; vda-ga@yandex.ru (D.A.V.); klever2023@mail.ru (N.S.T.); drsevastyan@yandex.ru (S.E.D.); genie@qsar.chem.msu.ru (E.V.R.); grishin@nmr.chem.msu.ru (Y.K.G.); vv1950@gmail.com (V.V.G.); 2 Institute of Physiologically Active Compounds at Federal Research Center of Problems of Chemical Physics and Medicinal Chemistry, Russian Academy of Sciences, Severny proezd 1, 142432 Chernogolovka, Moscow Region, Russia; av-post@mail.ru (A.V.G.); vzam@yandex.ru (V.L.Z.)

**Keywords:** heterocyclization, regioselective, tetranitromethane, isoxazole, bivalent ligand, AMPA receptor, PAM

## Abstract

An efficient regioselective approach to novel functionalized bis(isoxazoles) with a variety of aromatic and aliphatic linkers was elaborated, based on the heterocyclization reaction of electrophilic alkenes under the treatment with tetranitromethane-triethylamine complex affording 3-EWG-5-nitroisoxazoles. The subsequent *S_N_Ar* reactions of 5-nitroisoxazoles with various *O*,*O*-, *N*,*N*- and *S*,*S*-bis(nucleophiles) provide a wide range of bis(isoxazole) derivatives in good isolated yields. Employing an elaborated method, a series of novel bis(3-EWG-isoxazoles) as the promising allosteric modulators of AMPA receptors were designed and synthesized. The effect of the compounds on the kainate-induced currents was studied in the patch clamp experiments, revealing modulator properties for several of them. The best positive modulator potency was found for dimethyl 5,5′-(ethane-1,2-diylbis(sulfanediyl))bis(isoxazole-3-carboxylate), which potentiated the kainate-induced currents in a wide concentration range (10^−12^–10^−6^ M) with maximum potentiation of 77% at 10^−10^ M. The results were rationalized using molecular docking and molecular dynamics simulations of modulator complexes with the dimeric ligand-binding domain of the GluA2 AMPA receptor. The predicted physicochemical, ADMET, and PAINS properties confirmed that the AMPA receptor modulators based on the bis(isoxazole) scaffold may serve as potential lead compounds for the development of neuroprotective drugs.

## 1. Introduction

AMPA (α-amino-3-hydroxy-5-methyl-4-isoxazolepropionic acid) receptors are an important subtype of ionotropic glutamate receptors that play a crucial role in mediating fast synaptic transmission necessary for effective learning and memory formation [1,2]. Dysfunctions in the AMPA receptor activity are implicated in neurological and psychiatric disorders such as Alzheimer’s disease, epilepsy, depression, etc. [3,4,5,6,7]; therefore, AMPA receptors are promising targets for the development of cognitive enhancers [8]. It is known that positive allosteric modulators (PAMs) of the AMPA receptor, binding in its allosteric binding site, allow one to fine-tune the AMPA receptor signaling as well as to avoid the excitotoxicity problems commonly observed when a direct agonist is administered in excessive doses [9,10]. On the other hand, its negative allosteric modulators (NAMs) could have antiepileptic and neuroprotective properties [11,12,13,14].

Over the previous decades, massive efforts have been devoted to the search for novel AMPA receptor modulators with desired pharmacological efficacy and safety profiles [1,2,5,7,11,15,16]. Starting from the classical scaffolds such as benzamides, benzothiadiazines, biarylalkylsulfonamides, and trifluoromethylpyrazoles, many interesting PAM chemotypes have been identified. However, for a number of reasons, they have not yet reached clinical use, but this area of research is still considered valid and promising [11,17].

There is a particular interest in the bivalent ligands of the AMPA receptor because of their unique ability to bind simultaneously by two parts of the molecule at a close distance from each other in the pocket located at the interface between two subunits of the receptor [11,18,19]. Recently we reported several novel series of dimeric AMPA receptor PAMs based on different scaffolds; for many of them, potencies in the nanomolar or picomolar concentration ranges were demonstrated in the patch clamp experiments [20,21,22,23]. Amazingly, in many cases substantial differences in activity are found even for very similar structures, e.g., one structure could be a potent PAM while another is a potent NAM [11]. Such activity cliffs present an interesting but significant challenge in terms of understanding the mechanisms of action and the structure-activity relationships of the compounds. In order to enable in-depth analysis as well as to identify promising leads for pharmaceutical development, a thorough exploration of the modulator chemical space is required that needs to be supported by chemo- and regioselective synthetic approaches.

One of the promising heterocyclic cores for the structure of bivalent AMPA ligands is the isoxazole ring [11,22]. There are two main approaches to isoxazole ring formation consisting in (1) heterocyclization of 1,3-dielectrophiles, such as 1,3-diketones, 1,3-ketoesters, 1,3-ketoenamines, α,β-unsaturated ketones and others, under the treatment with hydroxylamine or (2) in reactions of 1,3-dipolar cycloaddition of nitrile oxides to acetylenes (or the reactions of their synthetic equivalents) [24,25,26,27,28,29,30]. However, both methods are not completely chemo- and regioselective, and many efforts have been devoted to improvement of their selectivity [31,32,33,34,35,36]. The direct functionalization of the isoxazole ring also has peculiar features and limitations. In general, the introduction of functional groups and substituents into the isoxazole fragment is carried out using transition metal catalysts, such as palladium and gold, in order to avoid highly acidic or basic conditions that destroy isoxazole derivatives [37,38,39,40,41].

Earlier, we have developed a novel preparative approach to 3-EWG-5-nitroisoxazoles involving the heterocyclization reaction of electrophilic alkenes with tetranitromethane-triethylamine (TNM-TEA) complex [42]. We also employed this method for further chemo- and regioselective functionalization of isoxazole moiety via the nitro group reduction [43] and aromatic nucleophilic substitution of nitro group with variety of nucleophiles [44]. These approaches allowed us to obtain the isoxazole derivatives with antiviral [45], antioxidant [46], and anticancer [47] activities. 

**Figure 1:**
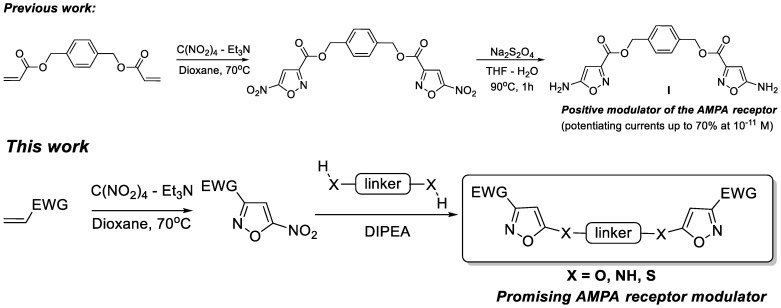
General approach to bis(isoxazole) AMPA receptor modulators.

In a recent publication, we reported the synthesis of bis(5-aminoisoxazole) **I** by the heterocyclization reaction of electrophilic diene with TNM-TEA complex and subsequent reduction of nitro groups of bis(5-nitroisoxazole) (Figure 1) [22]. The bis(heterocycle) **I** was shown to be a highly potent positive modulator of the AMPA receptor that potentiated the kainate-induced ion currents by up to 70% at the concentration of 10^−11^ M [22]. This result inspired us to examine another type of bis(isoxazoles) obtained by the reaction of 5-nitroisoxazoles with *O*,*O*-, *N*,*N*-, and *S*,*S*-bis(nucleophiles) containing different linkers (Figure 1). 

Herein, we report the development and optimization of synthetic approaches to these compounds and the in vitro evaluation of their AMPA receptor modulator activities. The structure-activity relationships in this series were analyzed using molecular modeling techniques such as molecular docking and molecular dynamics simulations, and the computational evaluation of their physicochemical, ADMET, and PAINS properties was performed to confirm the suitability of the AMPA receptor modulators of this type as potential lead compounds for the development of novel neuroprotective drugs.

## 2. Results and Discussion

### 2.1. Chemistry

The starting 3-EWG-5-nitroisoxazoles **1a**–**d** were obtained in good yields using the standard protocol for heterocyclization reaction of α,β-unsaturated esters and amides under the treatment with TNM-TEA complex [42]. Variation of substituents in position 3 of the isoxazole ring provides the structures of different sizes and levels of hydrophobicity.


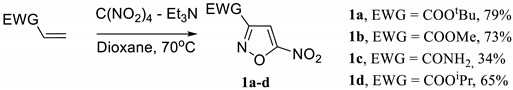


In order to investigate the influence of the linker nature on the modulating properties of bis(isoxazoles) to the AMPA receptor, the compounds **3a**–**c** with aromatic hydroquinone linker as well as the series of heterocycles **3d**–**j** bearing acyclic diamines and disulfides with aliphatic chains of various lengths were obtained.

We began our study with the *S_N_Ar* reaction of 5-nitroisoxazoles **1a**–**c** with hydroquinone as *O*,*O*-bis(nucleophile). It was found that 5-nitroisoxazoles **1a**,**b** with ester groups in position 3 of the isoxazole ring smoothly and regioselectively react with hydroquinone under the previously found conditions in acetonitrile at room temperature [44] affording bis(isoxazoles) **3a**,**b** in good yields (Table 1). However, the reaction of amide **1c** with hydroquinone in these conditions appeared incomplete due to the low solubility of the starting compound in acetonitrile at room temperature. So, bis(isoxazole) **3c** was isolated in 74% yield when the reaction proceeded at reflux in acetonitrile for 6 h.

It was found that the reactions of 5-nitroisoxazoles with aliphatic diamines and disulfides under the above-mentioned conditions lead to complex mixtures of products; therefore, additional optimization of conditions was required. The reaction between model 5-nitroisoxazole **1a** and 1,3-propanediamine as a bis(nucleophile) was studied by varying bases, solvents, reaction time, and temperature. When the reaction was performed in acetonitrile, the product **3e** was obtained in low yields regardless of the bases used as well as reaction time. Also, it was found that a slight increase in the yield of bis(isoxazole) **3e** was achieved when the temperature was increased in CH_3_CN or if THF was used as a solvent. When the reaction was carried out in EtOH, bis(isoxazole) **3e** was obtained in moderate yield (36%) because 5-nitroisoxazole partly reacted with EtOH to give 5-ethoxyisoxazole as a by-product. We have found that the yield of compound **3d** was significantly improved by changing the solvent in the *S_N_Ar* reaction to *tert*-BuOH, which can be explained by the low nucleophilicity of this solvent.

Thus, using the modified conditions, a series of bis(isoxazoles) **3d**–**j** bearing the *N*,*N*- and *S*,*S*- linear linkers were obtained in good yields as a single regioisomer in each case (Table 2). All synthesized compounds were identified and characterized using the ^1^H and ^13^C NMR spectroscopy.

### 2.2. Electrophysiological Evaluation and the Structure-Activity Relationships

All the synthesized compounds were examined for their AMPA receptor modulator activity in the electrophysiological experiments on freshly isolated Purkinje neurons using the patch clamp technique. Their influence on the kainate-induced currents is shown in Table 3. As can be seen, depending on the specific linkers and the substituents in the isoxazole core, the closely related compounds can sometimes have dramatically different activity profiles. Such activity cliffs are also often observed in other series of the bivalent ligands of the AMPA receptor [11].

Among the compounds based on the hydroquinone linker, the compound **3a** is a moderately potent negative potentiator of the AMPA receptor in the 10^−11^–10^−6^ M concentration range, causing a decrease in the current of approximately 17% at 10^−9^ M and 44% at 10^−6^ M. Two other compounds of this class (**3b**,**c**) with smaller isoxazole substituents are apparently inactive.

Among the compounds with diamine linkers, two compounds with longer linkers (**3e**,**f**) also were moderately potent negative modulators in this range, with the maximum decrease in the current of about 30–35% at 10^−9^–10^−8^ M. Interestingly, they have a bell-shaped concentration dependence that is rather unusual for the negative modulators. On the other hand, the compound **3d** with a shorter linker was a rather weak positive AMPA receptor modulator in the 10^−10^–10^−6^ M range with maximum potentiation (by up to 27%) at 10^−8^ M.

Finally, for the compounds based on the dithiol linkers (**3g**–**j**), the potentiation of the AMPA receptor currents was observed in a wide concentration range (up to 10^−12^–10^−6^ M) with a bell-shaped concentration dependence. Three of these are among the most potent known positive AMPA receptor modulators that have a maximum potentiation of 68% at 10^−9^ M (**3g**), 59% at 10^−8^ M (**3h**) and 77% at 10^−10^ M (**3j**). They are comparable with the previously reported reference compound **I** with a different isoxazole substitution pattern. 

### 2.3. Molecular Modeling

In order to clarify the likely mechanisms of action of the allosteric modulators **3a**–**j**, we used molecular docking and molecular dynamics simulations to study their interactions with the dimeric ligand-binding domain (LBD) of the GluA2 AMPA receptor. The visual inspection of the trajectories, as well as the plots of the root-mean-square deviations (RMSD) for the protein, glutamate and ligand heavy atoms, show that the systems retained stability and the compounds were stably bound in the PAM binding site at the interface between the LBD subunits over the entire course of the simulation (100 ns), although their exact positions often were adjusted compared to the initial docking poses (see Supplementary Materials, Figures S1 and S2).

**Figure 2:**
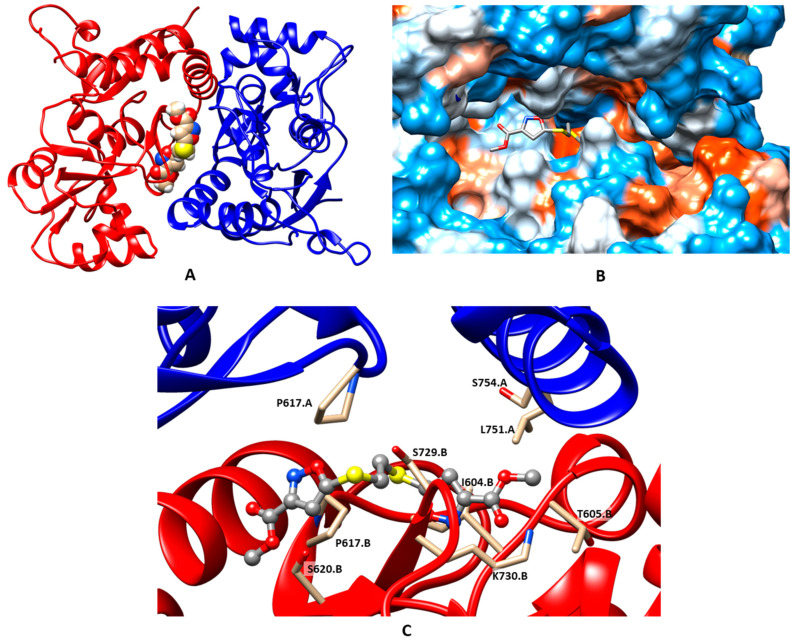
Binding mode of the PAM **3j**, refined using molecular dynamics simulation (100 ns). (**A**) General view of the dimeric ligand binding domain of AMPA receptor (GluA2) and location of the binding site. (**B**) Binding pockets in the protein molecular surface colored by local hydrophobicity (brown for hydrophobic and blue for hydrophilic). (**C**) Detailed view of the binding site. The ligand is represented by a grey ball-and-stick model, and the amino acid residues located within 3 Å of it are represented by beige stick models.

**Figure 3:**
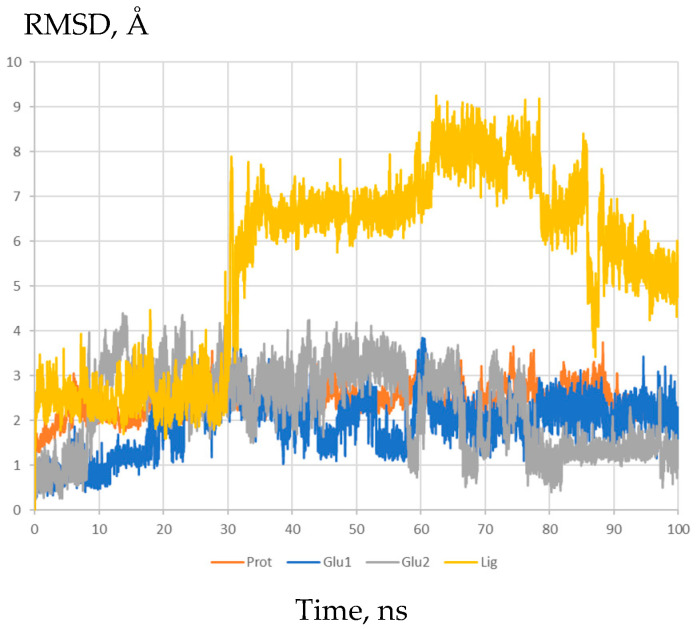
RMSD of the protein, glutamate and ligand **3j** heavy atoms during molecular dynamics simulation of the modulator complex with the dimeric ligand-binding domain of the GluA2 AMPA receptor.

In agreement with both the X-ray and molecular modeling data [11,18,19,20,22,23,48,49,50,51] for other larger and dimeric (“Class 3” [48]) modulators, the modulator molecules are located in the symmetrical PAM binding site, occupying its central subpocket as well as one or both of the side subpockets (Figure S1). As an example, the binding mode of the most potent PAM molecule **3j** is examined in detail in Figure 2. The binding was primarily stabilized by steric fit and hydrophobic interactions. The RMSD plots (Figure 3) confirm the stability of the system and demonstrate the adjustment of the ligand position.

However, as is often the case in series of closely related ligands acting on targets with complex structures and mechanisms of operation [52], no simple explanation of the differences in activity profiles for compounds **3a**–**j** could be derived from the inspection of their binding positions, RMSD plots, or binding free energies calculated using the MM/GBSA (molecular mechanics, generalized Born, surface area) method (see Supplementary Materials, Figures S1 and S2, Table S1). Previously, we have hypothesized that the negative allosteric modulator activity of the compounds could be mediated by their action on another target or binding site, such as the NAM binding site at the interface between the ligand-binding and transmembrane domains of the AMPA receptor [23]. Similar to the analysis [52] and inspired by visual observations of changes in the protein subunit arrangement over the course of the trajectories, we suggest that different PAM activity profiles could be related to finer differences in the subunit dynamics that could, in turn, affect the receptor gating and desensitization [1,11,53,54].

**Figure 4:**
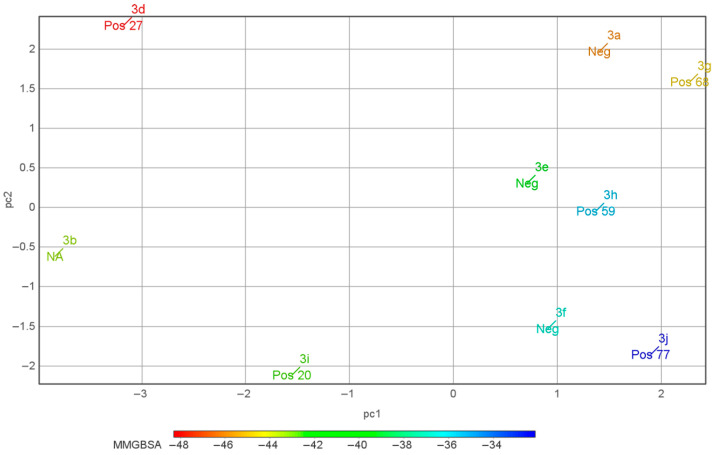
PCA plot showing the modulator activities of the compounds in the principal component space based on the MM/GBSA binding energies and the geometrical parameters of the subunit arrangement in their complexes with the AMPA receptor LBD. Pos—PAM, Neg—NAM, NA—no activity; for the PAMs, the potentiation percentage is also shown.

In order to evaluate this hypothesis, we tried to correlate the modulator activity profiles to their MM/GBSA binding energies and the geometrical parameters of the subunit arrangement (time-averaged angles and distances between their key secondary structure elements as explained in Section 3.3) using the principal component analysis (PCA). The values of the parameters are presented in the Supplementary Materials (Table S1). Compound **3c** was excluded to avoid distortion of the PCA model (its parameters are significantly different from the rest of the dataset), also taking into account its lack of activity. The plot of the compound positions in the PCA space (Figure 4) indicates that in this series, the highly potent PAMs (and, to a somewhat lesser extent, NAMs) of the AMPA receptor tend to cluster in the right part of the PCA plot (at high positive values of the first component), while moderately active PAMs and inactive compounds are found in the left part (at negative values of the first component). While this preliminary result is encouraging, in order to derive a reliable predictive model of PAM activity, a more detailed analysis is clearly required that should involve a broader and more diverse set of compounds as well as expand and refine the set of structural parameters.

### 2.4. Prediction of Physicochemical, ADMET, and PAINS Profiles

Several key physicochemical and ADMET properties for compounds **3a**–**j** were evaluated using predictive models (Table 4). High predicted values for intestinal absorption indicate their suitability for oral administration. According to the commonly accepted rules of thumb for potential drug-like compounds, the predicted lipophilicities and aqueous solubilities also were appropriate. Acceptable CNS bioavailability could be anticipated due to the moderate predicted blood–brain barrier permeability. For the cardiac toxicity risk, both predicted parameters (hERG p*K_i_* and pIC_50_, 4.3–7.4 log units) were in the lower or medium parts of their possible ranges (3–9 log units), indicating a likely absence of hERG liabilities. The integral quantitative estimate of drug-likeness (QED) was greater than 0.4, confirming the favorable likely properties. The pan-assay interference compounds (PAINS) filter check did not identify any alerts.

Overall, the predicted ADMET, physicochemical, and PAINS properties of the allosteric modulators **3a**–**j** were quite acceptable for the potential lead compounds at the early drug development stages, although additional checks and structure optimization would likely be required.

## 3. Materials and Methods

### 3.1. Chemistry

#### 3.1.1. General Remarks

The ^1^H (400 MHz) and ^13^C (101 MHz) NMR spectra were recorded on the Bruker Avance 400 spectrometer at ambient temperature. The chemical shifts δ were measured in ppm with respect to the solvent CDCl_3_ (^1^H: δ = 7.26 ppm, ^13^C: δ = 77.16 ppm), CD_3_OD (^1^H: δ = 3.31 ppm, ^13^C: δ = 49.00 ppm), and DMSO-*d*_6_ (^1^H: δ = 2.50 ppm, ^13^C: δ = 39.52 ppm). Chemical shifts (δ) are given in ppm; J values are given in Hz. Accurate molecular mass measurements were performed by HRMS using Bruker micrOTOF II instrument with electrospray ionization (ESI) in a positive ion mode (interface capillary voltage 4500 V) or a negative ion mode (3200 V). Melting points (mp) are uncorrected. Analytical thin-layer chromatography was performed using ALUGRAM silica gel plates (supported on aluminum) with detection by UV lamp (254 and 365 nm) and chemical staining (5% aqueous solution of KMnO_4_). Column chromatography was carried out on Macherey–Nagel silica gel 60 (0.040–0.063 mm).

5-Nitroisoxazoles **1a**–**c** [42] were synthesized by described methods. 

All other starting materials were commercially available.

All reagents except commercial products of satisfactory quality were purified by literature procedures prior to use.

*Isopropyl* 5-*nitroisoxazole*-3-*carboxylate* (**1d**) was obtained from isopropyl acrylate by our method using the complex of tetranitromethane and triethylamine [42]. Compound was isolated pure as colorless solid in 65% (0.65 g) yield; mp = 44–46 °C; R_f_ = 0.40 (petroleum ether:EtOAc = 10:1); ^1^H NMR (CDCl_3_, 400 MHz): δ 7.41 (s, 1H, CH), 5.36 (sept, ^3^J = 6.3 Hz, 1H, CH), 1.44 (d, ^3^J = 6.3 Hz, 6H, CH_3_); ^13^C NMR (CDCl_3_, 101 MHz): δ 21.8 (2CH_3_), 72.0 (CH), 102.5 (CH), 157.3 (C), 158.7 (C), 165.4 (br. C-NO_2_); HRMS-ESI (M + Na^+^): found: 223.0325. Calculated for C_7_H_8_N_2_O_5_Na^+^: 223.0320.

#### 3.1.2. Synthesis of Bis(isoxazoles) **3**

Bis(nucleophile) (0.2 mmol) and DIPEA (0.4 mmol, 52 mg, 0.070 mL) were added sequentially to the solution of 5-nitroisoxazole **1** (0.2 mmol) in CH_3_CN (3 mL) in case of hydroquinone or in ^t^BuOH (3 mL) in case of diamines or dithiols, and the resulting mixture was stirred at r.t. for 48 h (for compounds **3a**,**b**,**d**–**j**) or refluxed for 2 h (for compound **3c**). At the end of the reaction (controlled by TLC), the solvent was evaporated in vacuo to give the crude product, which was purified by column chromatography.

*Di*-*tert*-*butyl* 5,5′-(1,4-*phenylenebis*(*oxy*))*bis*(*isoxazole*-3-*carboxylate*) (**3a**) [44] was isolated pure as colorless solid in 74% (70 mg) yield; mp = 175–178 °C; R_f_ = 0.14 (petroleum ether:EtOAc = 4:1); ^1^H NMR (CDCl_3_, 400 MHz): δ 1.59 (s, 18H, 6CH_3_), 5.69 (s, 2H, 2CH), 7.27 (s, 4H, 4CH(Ar)); ^13^C NMR (CDCl_3_, 100.6 MHz): δ 28.1 (6CH_3_), 82.8 (2CH), 84.1 (2C), 120.9 (4CH), 151.9 (2C), 158.7 (2C), 159.3 (2C); HRMS-ESI (M + H^+^): found: 445.1598. Calculated for C_22_H_25_N_2_O_8_^+^: 445.1605.

*Dimethyl* 5,5′-(1,4-*phenylenebis*(*oxy*))*bis*(*isoxazole*-3-*carboxylate*) (**3b**) was isolated pure as colorless solid in 77% (55 mg) yield; mp = 136–138 °C; R_f_ = 0.14 (petroleum ether:EtOAc = 4:1); ^1^H NMR (CDCl_3_, 400 MHz): δ 3.96 (s, 6H, 2CH_3_), 5.77 (s, 2H, 2CH), 7.29 (s, 4H, 4CH(Ar)); ^13^C NMR (CDCl_3_, 100.6 MHz): δ 53.1 (2CH_3_), 82.8 (2CH), 121.1 (4CH(Ar)), 151.9 (2C), 157.9 (2C), 160.1 (2C), 172.7 (2C=O); HRMS-ESI (M + H^+^): found: 361.0666. Calculated for C_16_H_13_N_2_O_8_^+^: 361.0662.

5,5′-(1,4-*phenylenebis*(*oxy*))*bis*(*isoxazole*-3-*carboxamide*) (**3c**) was crystallized after the addition of MeOH into the reaction mixture and required no additional purification. Compound was isolated pure as yellowish solid in 85% (56 mg) yield; mp = 254–257 °C; ^1^H NMR (DMSO-*d*_6_, 400 MHz): δ 5.94 (s, 2H, 2CH), 7.53 (s, 4H, 4CH(Ar)), 7.84 (s, 2H, NH_2_), 8.09 (s, 2H, NH_2_); ^13^C NMR (DMSO-*d*_6_, 100.6 MHz): δ 81.5 (2CH), 121.4 (4CH(Ar)), 151.5 (2C), 159.8 (2C), 160.9 (2C), 172.0 (2C); HRMS-ESI (M + H^+^): found: 403.2013. Calculated for C_25_H_27_N_2_O_3_^+^: 403.2016.

*Di*-*tert*-*butyl* 5,5′-(*ethane*-1,2-*diylbis*(*azanediyl*))*bis*(*isoxazole*-3-*carboxylate*) (**3d**) was isolated pure as a colorless solid in 86% (68 mg) yield; mp = 163–165 °C; R_f_ = 0.41 (petroleum ether:EtOAc = 2:1); ^1^H NMR (CDCl_3_-CD_3_OD, 400 MHz): δ 1.55 (s, 18H, 6CH_3_), 2.15 (br.s, 2H, 2NH), 3.41 (s, 4H, 2CH_2_), 5.30 (s, 2H, 2CH); ^13^C NMR (CDCl_3_-CD_3_OD, 101 MHz): δ 28.1 (6CH_3_), 43.4 (2CH_2_), 78.0 (2CH), 83.5 (2CO), 158.5 (2C), 159.8 (2C), 170.8 (2C); HRMS-ESI (M + Na^+^): found: 417.1746. Calculated for C_18_H_26_N_4_O_6_Na^+^: 427.2380.

*Di*-*tert*-*butyl* 5,5′-(*propane*-1,3-*diylbis*(*azanediyl*))*bis*(*isoxazole*-3-*carboxylate*) (**3e**) was isolated pure as colorless solid in 77% (63 mg) yield; mp = 171–174 °C; R_f_ = 0.45 (petroleum ether:EtOAc = 1:1); ^1^H NMR (CDCl_3_, 400 MHz): δ 1.57 (s, 18H, 6CH_3_), 1.94 (quint, *J* = 6.5 Hz, 2H, CH_2_), 3.33–3.38 (m, 4H, 2CH_2_), 5.32 (s, 2H, 2CH), 5.44 (br.t, *J* = 6.1 Hz, 2H, 2NH); ^13^C NMR (CDCl_3_, 101 MHz): δ 28.2 (6CH_3_), 28.8 (CH_2_), 41.6 (2CH_2_), 77.9 (2CH), 83.4(2CO), 158.6 (2C), 159.9 (2C), 171.0 (2C); HRMS-ESI (M + NH_4_^+^): found: 427.2374. Calculated for C_19_H_32_N_5_O_6_^+^: 427.2380.

*Di*-*tert*-*butyl* 5,5′-(*butane*-1,4-*diylbis*(*azanediyl*))*bis*(*isoxazole*-3-*carboxylate*) (**3f**) was isolated pure as colorless solid in 88% (74 mg) yield; mp = 180–181 °C; R_f_ = 0.42 (petroleum ether:EtOAc = 2:1); ^1^H NMR (CDCl_3_, 400 MHz): δ 1.56 (s, 18H, 6CH_3_), 1.68–1.74 (m, 4H, 2CH_2_), 3.20–3.27 (m, 4H, 2CH_2_), 5.10 (br.t, *J =* 6.1 Hz, 2H, 2NH), 5.30 (s, 2H, 2CH); ^13^C NMR (CDCl_3_, 101 MHz): δ 26.8 (2CH_2_), 28.2 (6CH_3_), 44.2 (2CH_2_), 77.8 (2CH), 83.3 (2CO), 158.6 (2C), 159.9 (2C), 171.0 (2C); HRMS-ESI (M + Na^+^): found: 445.2049. Calculated for C_20_H_30_N_4_O_6_Na^+^: 445.2058.

*Di*-*tert*-*butyl* 5,5′-(*ethane*-1,2-*diylbis*(*sulfanediyl*))*bis*(*isoxazole*-3-*carboxylate*) (**3g**) was isolated pure as colorless oil in 68% (58 mg) yield; R_f_ = 0.48 (petroleum ether:EtOAc = 3:1); ^1^H NMR (CDCl_3_, 400 MHz): δ 1.60 (s, 18H, 6CH_3_), 3.32 (s, 4H, 2CH_2_), 6.56 (s, 2H, 2CH); ^13^C NMR (CDCl_3_, 101 MHz): δ 28.2 (6CH_3_), 33.5 (2CH_2_), 84.1 (2CO), 105.9 (2CH), 158.5 (2C), 158.6 (2C), 166.4 (2C); HRMS-ESI (M + NH_4_^+^): found: 466.1409. Calculated for C_18_H_28_N_3_O_6_S_2_^+^: 446.1414.

*Di*-*tert*-*butyl* 5,5′-(*propane*-1,3-*diylbis*(*sulfanediyl*))*bis*(*isoxazole*-3-*carboxylate*) (**3h**) was isolated pure as colorless oil in 73% (65 mg) yield; R_f_ = 0.66 (petroleum ether:EtOAc = 2:1); ^1^H NMR (CDCl_3_, 400 MHz): δ 1.59 (s, 18H, 6CH_3_), 2.08 (quint, *J =* 6.9 Hz, 2H, CH_2_), 3.17 (t, *J* = 6.9 Hz, 4H, 2CH_2_), 6.52 (s, 2H, 2CH); ^13^C NMR (CDCl_3_, 101 MHz): δ 28.1 (6CH_3_), 29.5 (CH_2_), 31.8 (2CH_2_), 84.0 (2CO), 105.3 (2CH), 158.3 (2C), 158.7 (2C), 167.4 (2C); HRMS-ESI (M + NH_4_^+^): found: 466.1567. Calculated for C_19_H_30_N_3_O_6_S_2_^+^: 460.1571.

*Diisopropyl* 5,5′-(*ethane*-1,2-*diylbis*(*sulfanediyl*))*bis*(*isoxazole*-3-*carboxylate*) (**3i**) was isolated pure as colorless oil in 55% (44 mg) yield; R_f_ = 0.37 (petroleum ether:EtOAc = 5:1); ^1^H NMR (CDCl_3_, 400 MHz): δ 1.39 (d, *J =* 6.3 Hz, 12H, 4CH_3_), 3.34 (s, 4H, 2CH_2_), 5.30 (sept, *J* = 6.3 Hz, 2H, 2CH), 6.62 (s, 2H, 2CH); ^13^C NMR (CDCl_3_, 101 MHz): δ 21.9 (4CH_3_), 33.5 (2CH_2_), 70.6 (2CH), 105.9 (2CH), 157.6 (2C), 159.1 (2C), 166.7 (2C); HRMS-ESI (M + K^+^): found: 439.0391. Calculated for C_16_H_20_KN_2_O_6_S_2_^+^: 439. 0394.

*Dimethyl* 5,5′-(*ethane*-1,2-*diylbis*(*sulfanediyl*))*bis*(*isoxazole*-3-*carboxylate*) (**3j**) was isolated pure as colorless solid in 68% (47 mg) yield; mp = 127–129 °C; R_f_ = 0.15 (petroleum ether:EtOAc = 3:1); ^1^H NMR (CDCl_3_, 400 MHz): δ 3.36 (s, 4H, 2CH_2_), 3.97 (s, 6H, 2CH_3_), 6.62 (s, 2H, 2CH); ^13^C NMR (CDCl_3_, 100.6 MHz): δ 33.4 (2CH_2_), 53.2 (2CH_3_), 105.7 (2CH), 157.0 (2C), 159.9 (2C), 167.0 (2C); HRMS-ESI (M + H^+^): found: 345.0211. Calculated for C_12_H_13_N_2_O_6_S_2_^+^: 345.0210.

### 3.2. Electrophysiological Evaluation

The AMPA receptor modulator activity of the compounds was evaluated in electrophysiological experiments in vitro using a patch clamp technique on the freshly isolated rat Purkinje neurons (for details, see Supplementary Materials, Section S2) as described earlier [20,22].

### 3.3. Molecular Modeling

The modeling of the ligand interactions with the dimeric ligand-binding domain of the GluA2 AMPA was performed as described earlier [22,23] (for detailed computational protocol, see Supplementary Materials, Section S3). In brief, upon clean up of the structure obtained from the Protein Data Bank (PDB: 4FAT) [55], the protein was allowed to relax during the molecular dynamics simulation, and the most frequently occurring structure over the stable part of the trajectory was identified. The protein and ligand structures were prepared for molecular docking using the Avogadro 1.2.0 (Avogadro Chemistry, https://avogadro.cc/ accessed on 1 September 2023) [56] and AutoDock Tools 1.5.7 (The Scripps Research Institute, La Jolla, CA, USA, https://ccsb.scripps.edu/mgltools/ accessed on 1 September 2023) [57] software. The molecular docking into the positive allosteric modulator binding site was performed with the AutoDock Vina 1.1.2 software (The Scripps Research Institute, La Jolla, CA, USA, https://vina.scripps.edu/ accessed on 1 September 2023) [58]. The complex model based on the best selected pose was built using the USCF Chimera 1.15 software (University of California San Francisco, San Francisco, CA, USA, https://www.cgl.ucsf.edu/chimera/ accessed on 1 September 2023) [59].

The molecular dynamics simulations were performed using the CHARMM36/CGenFF 4.6 force field [60,61] on the GROMACS 2023.0 software (GROMACS development team, https://www.gromacs.org/ accessed on 1 September 2023) [62]. The initial models of the systems were built using the CHARMM-GUI web service (https://charmm-gui.org/ accessed on 1 September 2023) [63,64]. For the analysis and visualization of the results, the CPPTRAJ 6.4.4 software (Daniel R. Roe, Amber development team, http://ambermd.org/ accessed on 1 September 2023) [65] in the AmberTools 22 package [66] and UCSF Chimera were used. The binding free energies were estimated using the MM/GBSA approach implemented using the gmx_MMPBSA 1.6.1 software (gmx_MMPBSA development team, https://valdes-tresanco-ms.github.io/gmx_MMPBSA/dev/ accessed on 1 September 2023) [67,68].

**Figure 5:**
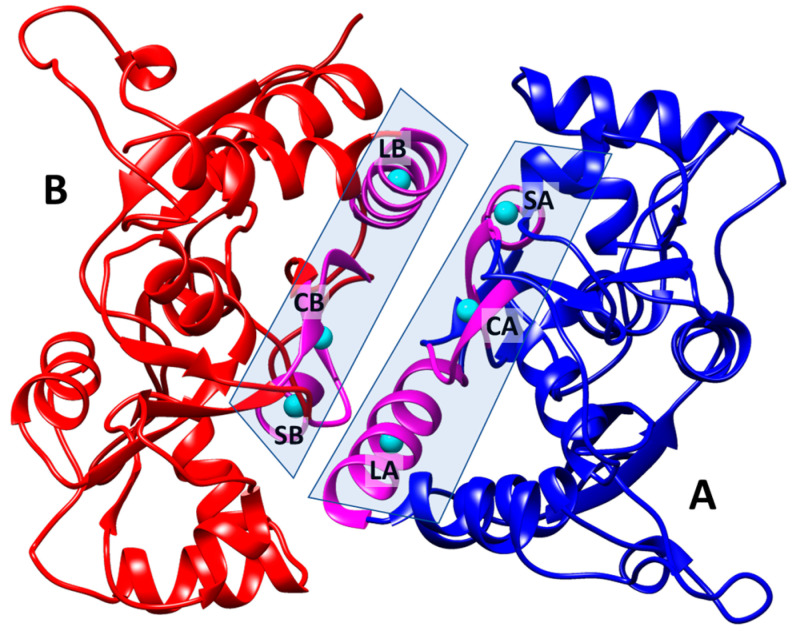
Definitions of key secondary structure elements for the analysis of LBD subunit arrangement. At the interface in both subunits (A and B), the centroids are defined for the Cα atoms of the small (S, residues 606–610) and large (L, residues 742–756) α-helices as well as for the central β-sheet (C, residues 617–622, 727–732). The secondary structure elements are shown in magenta and the centroids in cyan. The three centroids define the interface plane for each subunit.

For the analysis of the ligand influences on the LBD subunit arrangement dynamics, a number of geometrical parameters (angles and distances between the key secondary structure elements at the interface of subunits A and B) were defined as shown in Figure 5 and Table 5. The parameters were calculated with a Python script using the PYTRAJ 2.0.6 software (Hai Nguyen, Daniel R. Roe, Jason Swails, David A. Case, Amber development team, https://github.com/Amber-MD/pytraj/ accessed on 1 September 2023) [65,69] in the AmberTools 22 package [66] and their average values over the stable portion of the trajectories (last 20 ns, 101 frames at 200 ps interval) listed in Table S1 were used to build the PCA models.

DataWarrior 5.5.0 software (Idorsia Pharmaceuticals Ltd., https://openmolecules.org/ accessed on 1 September 2023) was used for the management, search, and analysis of the structure–activity data.

### 3.4. Prediction of Physicochemical, ADMET, and PAINS Profiles

The lipophilicity (LogP_ow_) and aqueous solubility (pS_aq_) were estimated using the OCHEM platform [70] and the ALogPS 3.0 neural network model. The integrated online service for ADMET properties prediction (ADMET Prediction Service) [71] was employed to predict the human intestinal absorption (HIA) [72], blood–brain barrier permeability (LogBB) [73], and hERG-mediated cardiac toxicity risk (channel affinity p*K_i_* and inhibitory activity pIC_50_) [74]. The quantitative estimate of drug-likeness (QED) values [75] were calculated and the pan-assay interference compounds (PAINS) alerts were checked using RDKit version 2020.03.4 software [76].

## 4. Conclusions

We have elaborated an efficient, simple and concise approach to the previously unknown functionalized bis(isoxazoles) bearing aromatic and aliphatic linkers with *O*,*O*-, *N*,*N*- or *S*,*S*-nucleophilic centers that involves the heterocyclization reaction of readily available electrophilic alkenes upon the treatment with TNM-TEA complex and subsequent regioselective aromatic nucleophilic substitution of the nitro group in a resulting 5-nitroisoxazole with variety of bis(nucleophiles). The reaction allows the linkers of different natures and tolerates a variety of moieties in the isoxazole ring. The remarkable activity of bis(isoxazoles) as positive allosteric modulators of the AMPA receptor was demonstrated in the electrophysiological experiments. The potentiation of the kainate-induced AMPA receptor currents was observed in a wide concentration range (10^−12^–10^−6^ M), and three of the compounds bearing the dithiol linker were among the most potent known AMPA receptor PAMs with the maximum potentiation of 68% at 10^−9^ M (**3g**), 59% at 10^−8^ M (**3h**) and 77% at 10^−10^ M (**3j**). Several other compounds also were found to be moderately potent negative modulators of the receptor, forming interesting activity cliffs. 

Molecular modeling confirms that these compounds can interact with the validated PAM binding site. However, as is often the case in the series of closely related ligands acting on targets with complex structures and mechanisms of operation, no simple correlation or explanation of the differences in activity profiles for compounds **3a**–**j** could be derived from the inspection of their binding positions or estimated free energies. Nevertheless, a multivariate analysis of finer LBD subunit dynamics parameters leads to a rough correlation. While this preliminary result is encouraging, in order to derive a reliable predictive model of PAM activity, a more detailed analysis is clearly required that should involve a broader and more diverse set of compounds as well as expand and refine the set of structural parameters. 

The predicted physicochemical, ADMET, and PAINS properties of the bis(isoxazoles) **3a**–**j** are quite acceptable for potential lead compounds at the early drug development stages. Taking into account the synthetic accessibility and possibility of the “property fine-tuning” for the heterocycles of this structural type, we can envision their further beneficial applications in the design of novel promising compounds possessing neuroprotective and nootropic activities.

## Figures and Tables

**Table 1:** Optimization of the reaction between 5-nitroisoxazole **1a** and diamine **2e**.

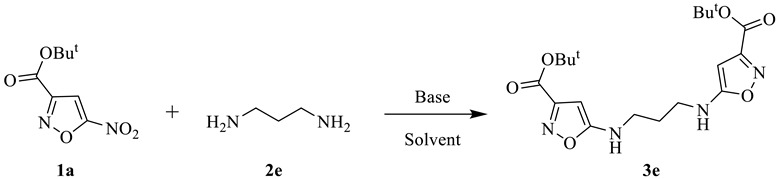					
**N**	**Base**	**Solvent**	**Reaction Time**	**Temperature**	**Yield 3e, %**
1	Cs_2_CO_3_	CH_3_CN	7 days	20 °C	5
2	K_2_CO_3_	CH_3_CN/H_2_O	48 h	20 °C	5
3	DIPEA	CH_3_CN	96 h	20 °C	-
4	DIPEA	CH_3_CN	2 h	80 °C	16
5	Et_3_N	THF	48 h	20 °C	15
6	DIPEA	EtOH	48 h	20 °C	36
7	DIPEA	^t^BuOH	48 h	20 °C	77

Note: Reactions of **1a** (0.2 mmol) and 1,3-propanediamine (0.1 mmol) in the presence of a base (0.2 mmol) were carried out in 3 mL of solvent.

**Table 2:** Nucleophilic substitution reaction results between 5-nitroisoxazoles **1a**–**d** and various *O*,*O*-, *N*,*N*-, and *S*,*S*-bis(nucleophiles).

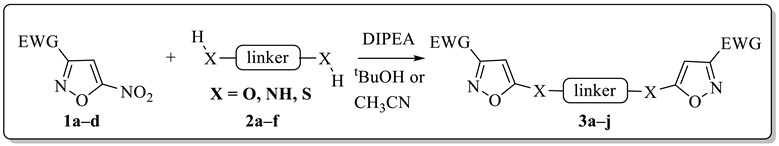			
**Compound**	**EWG**	**X-Linker-X**	**Yield**
**3a**	COO^t^Bu	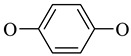	84%
**3b**	COOMe	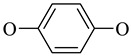	85%
**3c**	CONH_2_	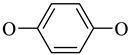	74%
**3d**	COO^t^Bu	NHCH_2_CH_2_NH	86%
**3e**	COO^t^Bu	NHCH_2_CH_2_CH_2_NH	77%
**3f**	COO^t^Bu	NHCH_2_CH_2_CH_2_CH_2_NH	88%
**3g**	COO^t^Bu	SCH_2_CH_2_S	68%
**3h**	COO^t^Bu	SCH_2_CH_2_CH_2_S	73%
**3i**	COO^i^Pr	SCH_2_CH_2_S	55%
**3j**	COOMe	SCH_2_CH_2_S	68%

Note: Reactions of **1a**–**c** (0.4 mmol) and hydroquinone (0.2 mmol) in the presence of DIPEA (0.4 mmol) were carried out in 3 mL of CH_3_CN at r.t. for 48 h (**1a**,**b**) or under reflux for 2 h (**1c**). Reactions of **1a**–**d** (0.4 mmol) and diamines (0.2 mmol) or dithiols (0.2 mmol) in the presence of DIPEA (0.4 mmol) were carried out in 3 mL of ^t^BuOH at r.t. for 48 h.

**Table 3:** The effect of various concentrations of the compounds on the kainate-induced AMPA receptor currents in rat cerebellum Purkinje cells.

Compound	Number of Neurons	Compound Concentration (M) and Current Amplitude (% to Control ± SD)						
		10^−12^	10^−11^	10^−10^	10^−9^	10^−8^	10^−7^	10^−6^
**3** **a**	5	–	96 ± 4	90 ± 5	83 ± 4	79 ± 5	76 ± 5	56 ± 5
**3** **b**	5	–	–	100 ± 1	100 ± 3	100 ± 2	100 ± 2	100 ± 3
**3** **c**	5	–	–	100 ± 1	100 ± 3	100 ± 2	100 ± 2	100 ± 3
**3d**	3	100 ± 3	100 ± 3	106 ± 4	120 ± 6	127 ± 6	119 ± 5	103 ± 3
**3e**	3	100 ± 2	100 ± 3	89 ± 4	77 ± 6	71 ± 5	88 ± 4	97 ± 3
**3f**	3	100 ± 4	95 ± 4	88 ± 6	78 ± 6	86 ± 5	92 ± 4	99 ± 2
**3g**	4	125 ± 5	**147 ± 6**	**156 ± 7**	**168 ± 7**	134 ± 4	113 ± 4	105 ± 3
**3h**	3	100 ± 2	116 ± 4	**143 ± 5**	**153 ± 5**	**159 ± 6**	**142 ± 5**	121 ± 4
**3i**	4	100 ± 3	117 ± 4	118 ± 5	118 ± 5	120 ± 6	122 ± 6	109 ± 5
**3j**	3	100 ± 3	**139 ± 8**	**177 ± 10**	**163 ± 11**	**146 ± 8**	141 ± 10	138 ± 9
**I** [22]	4	141 ± 7	172 ± 9	152 ± 7	144 ± 5	129 ± 4	113 ± 4	105 ± 3
**CTZ**	8						100 ± 3	145 ± 11

Note: The range of maximum potentiation is marked in bold.

**Table 4:** Predicted physicochemical and ADMET profiles of compounds **3a**–**j**.

Compound	MW	LogP_ow_	pS_aq_	LogBB	HIA	hERG p*K_i_*	hERG pIC_50_	QED
**3a**	402.50	5.11	6.63	−0.34	84	5.37	4.33	0.60
**3b**	430.55	5.75	6.91	−1.43	84	5.64	4.36	0.50
**3c**	458.61	5.93	7.13	−0.29	93	5.37	4.63	0.40
**3d**	486.66	6.09	7.89	−0.27	100	6.35	4.41	0.39
**3e**	454.57	5.48	7.08	−0.23	93	5.78	4.56	0.43
**3f**	374.44	4.36	5.49	−1.60	84	5.37	4.59	0.66
**3g**	374.44	4.36	5.77	−0.40	84	5.13	4.57	0.67
**3h**	430.55	5.64	7.23	−0.28	84	5.39	4.64	0.47
**3i**	406.53	5.01	6.59	0.22	100	7.37	4.69	0.52
**3j**	434.50	2.37	2.96	−0.53	84	5.09	4.48	0.46

Note: MW—molecular weight; LogP_ow_—octanol–water partition coefficient; pS_aq_—aqueous solubility [−log(M)]; LogBB—blood–brain barrier permeability; HIA—human intestinal absorption [%]; hERG p*K_i_*—hERG potassium channel affinity [−log(M)]; hERG pIC_50_—hERG potassium channel inhibitory activity [−log(M)]; QED—quantitative estimate of drug-likeness.

**Table 5:** Definitions of the geometrical parameters of the LBD subunit arrangement.

Name	Description
angleAB	Angle between the A and B planes (0 = parallel)
heightFaceAB	Mean height of the plane center point over the other plane
distFace	Distance between the face center points
shiftFaceAB	Mean lateral shift between the face center point and the projection of the other face center point
heightS_AB	Mean height of the plane S point over the other plane
shiftSL_AB	Mean lateral shift between the projection of the face S point and the other face L point
heightL_AB	Mean height of the plane L point over the other plane
shiftLS_AB	Mean lateral shift between the projection of the face L point and the other face S point

## Data Availability

Data are contained within the article.

## Supplementary Materials

The supporting information can be downloaded at: https://www.mdpi.com/article/10.3390/ijms242216135/s1.
