# Supplementary material for: 5-Nitroisoxazoles in SNAr Reactions: A Novel Chemo- and Regioselective Approach to Isoxazole-Based Bivalent Ligands of AMPA Receptors
Source: Int J Mol Sci. 2023 Nov 9;24(22):16135. doi: 10.3390/ijms242216135 (PMC10671298; doi:10.3390/ijms242216135)
Supplement: Supplementary file 1 [file ijms-24-16135-s001.zip › ijms-2689132-supplementary.pdf]

International Journal of
Molecular Sciences

MDPI

Article

5-Nitroisoxazoles in *SNAr* Reactions: A Novel Chemo- and Regioselective Approach to Isoxazole-Based Bivalent Ligands of AMPA Receptors

Dmitry A. Vasilenko [1], Nadezhda S. Temnyakova [1], Sevastian E. Dronov [1], Eugene V. Radchenko [1], Yuri K. Grishin [1], Alexey V. Gabrel'yan [2], Vladimir L. Zamoyski [2], Vladimir V. Grigoriev [1,2], Elena B. Averina [1,*] and Vladimir A. Palyulin [1,*]

[1] Department of Chemistry, Lomonosov Moscow State University, Leninskie Gory 1/3, 119991 Moscow, Russia; vda-ga@yandex.ru (D.A.V.); klever2023@mail.ru (N.S.T.); drsevastyan@yandex.ru (S.E.D.); genie@qsar.chem.msu.ru (E.V.R.); grishin@nmr.chem.msu.ru (Y.K.G.); vv1950@gmail.com (V.V.G.)

[2] Institute of Physiologically Active Compounds at Federal Research Center of Problems of Chemical Physics and Medicinal Chemistry, Russian Academy of Sciences, Severny proezd 1, 142432 Chernogolovka, Moscow Region, Russia; av-post@mail.ru (A.V.G.); vzam@yandex.ru (V.L.Z.)

Supplementary Materials

S1. NMR Spectra of Compounds 1d, 3b–j 2

S2. Electrophysiological Evaluation 22

S3. Molecular Modeling 23

Figure S1 24

Figure S2 25

Table S1 26

References 27

Isopropyl 5-nitroisoxazole-3-carboxylate **1d** (^{1}H NMR)

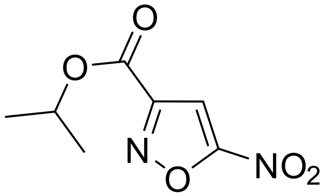

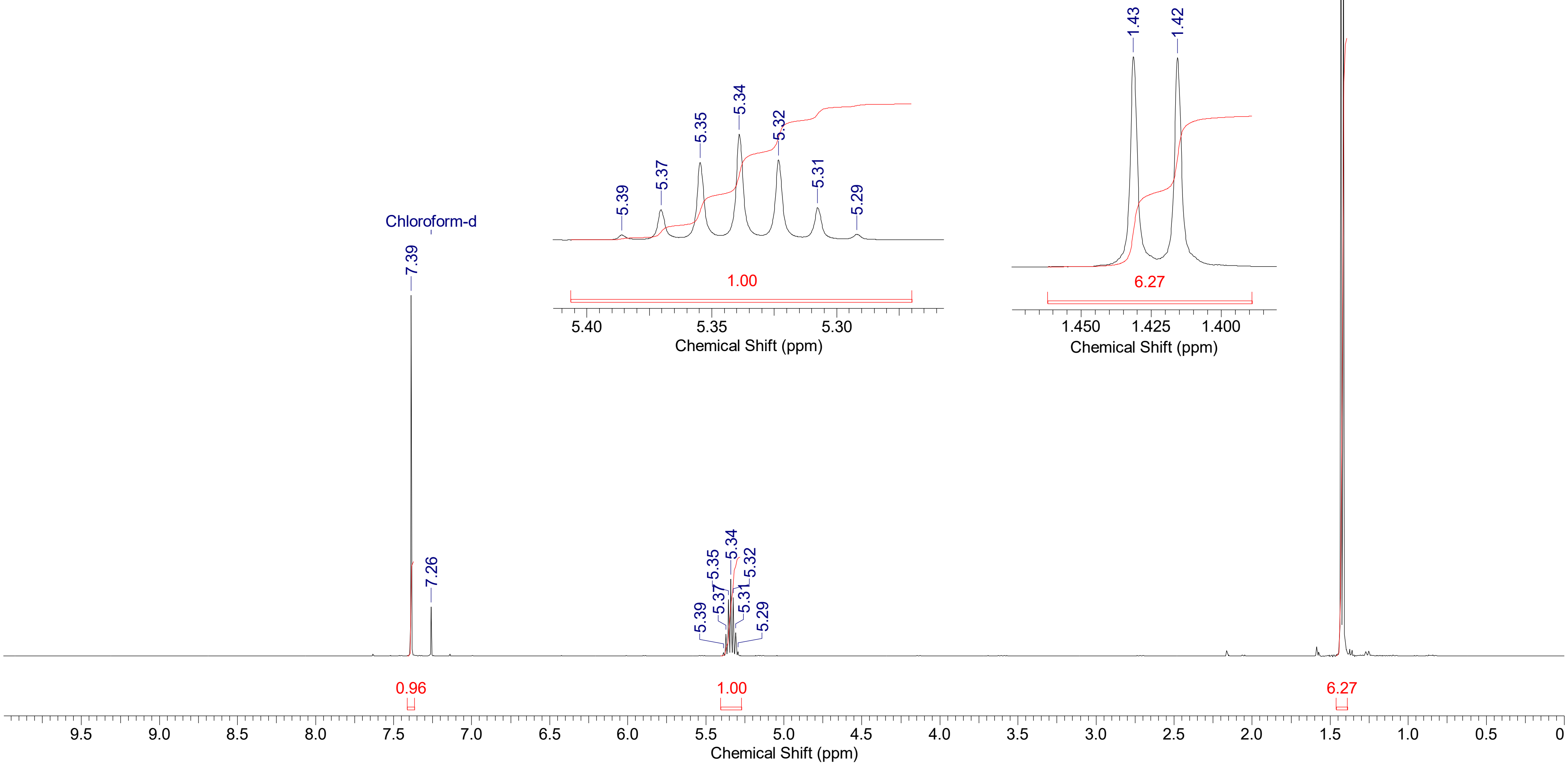

Isopropyl 5-nitroisoxazole-3-carboxylate **1d** (^{13}C NMR)

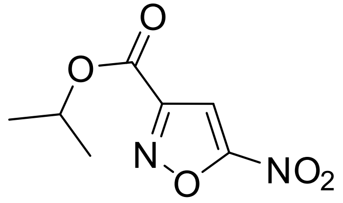

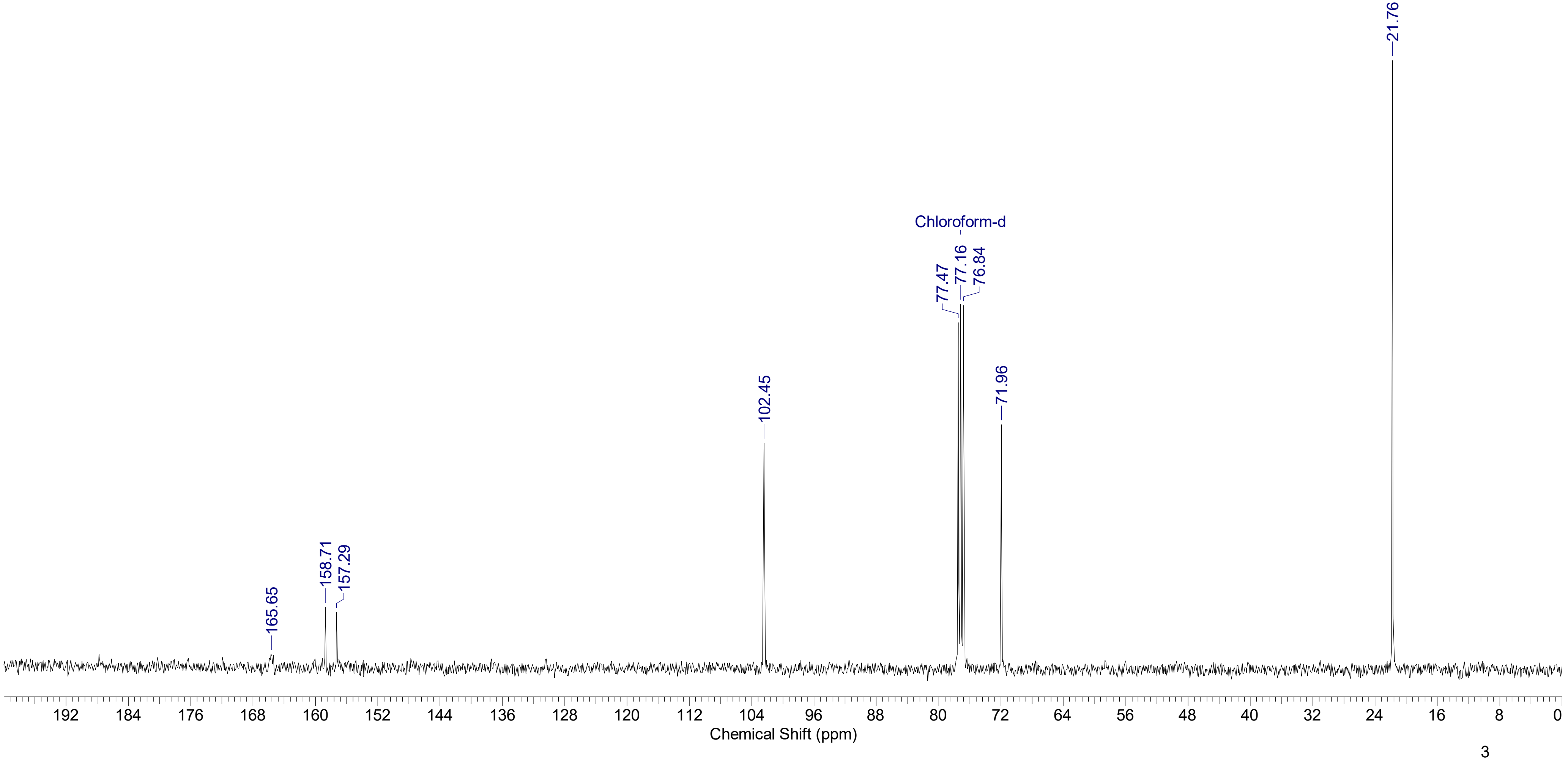

Dimethyl 5,5'-(1,4-phenylenebis(oxy))bis(isoxazole-3-carboxylate) **3b** (^{1}H NMR)

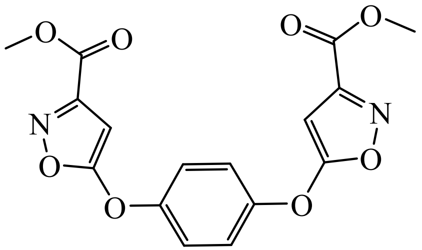

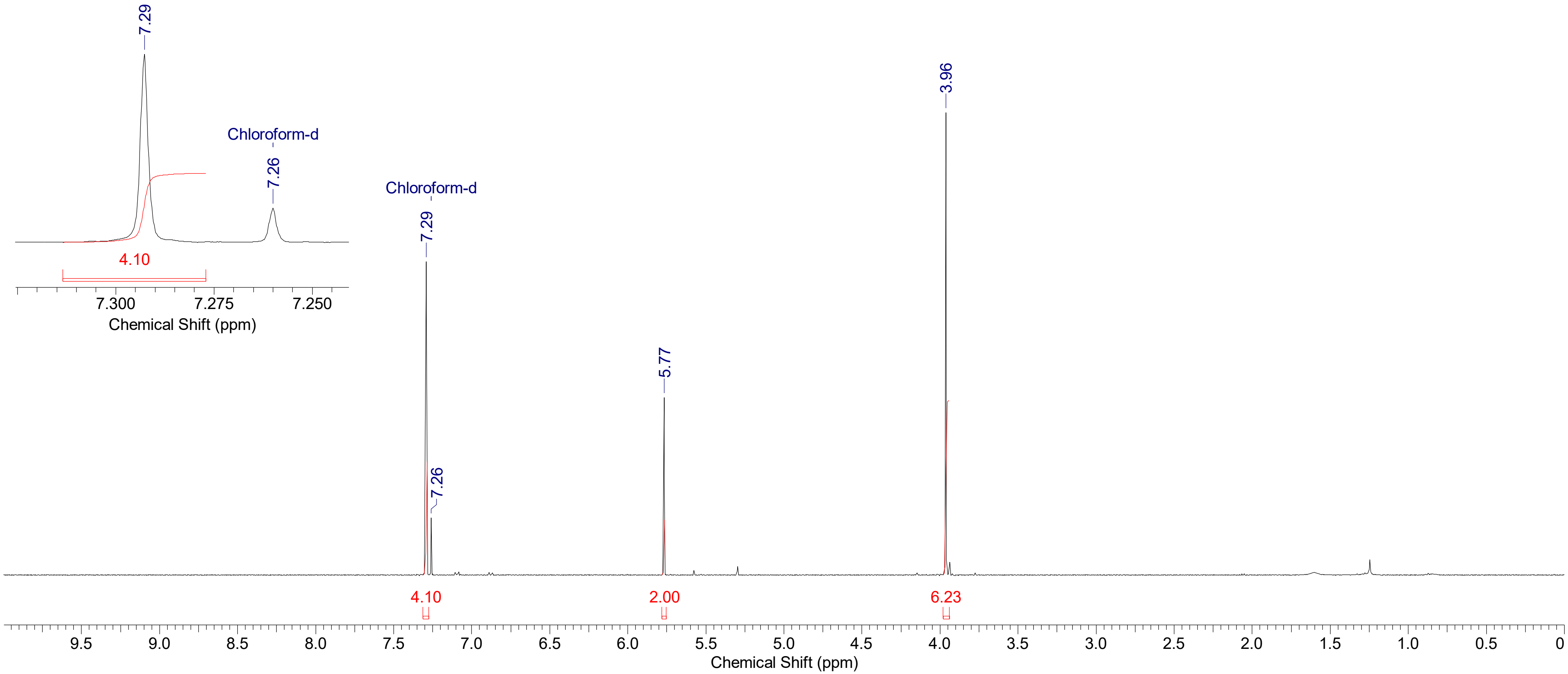

Dimethyl 5,5'-(1,4-phenylenebis(oxy))bis(isoxazole-3-carboxylate) **3b** (^{13}C NMR)

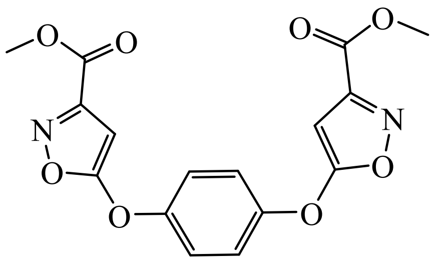

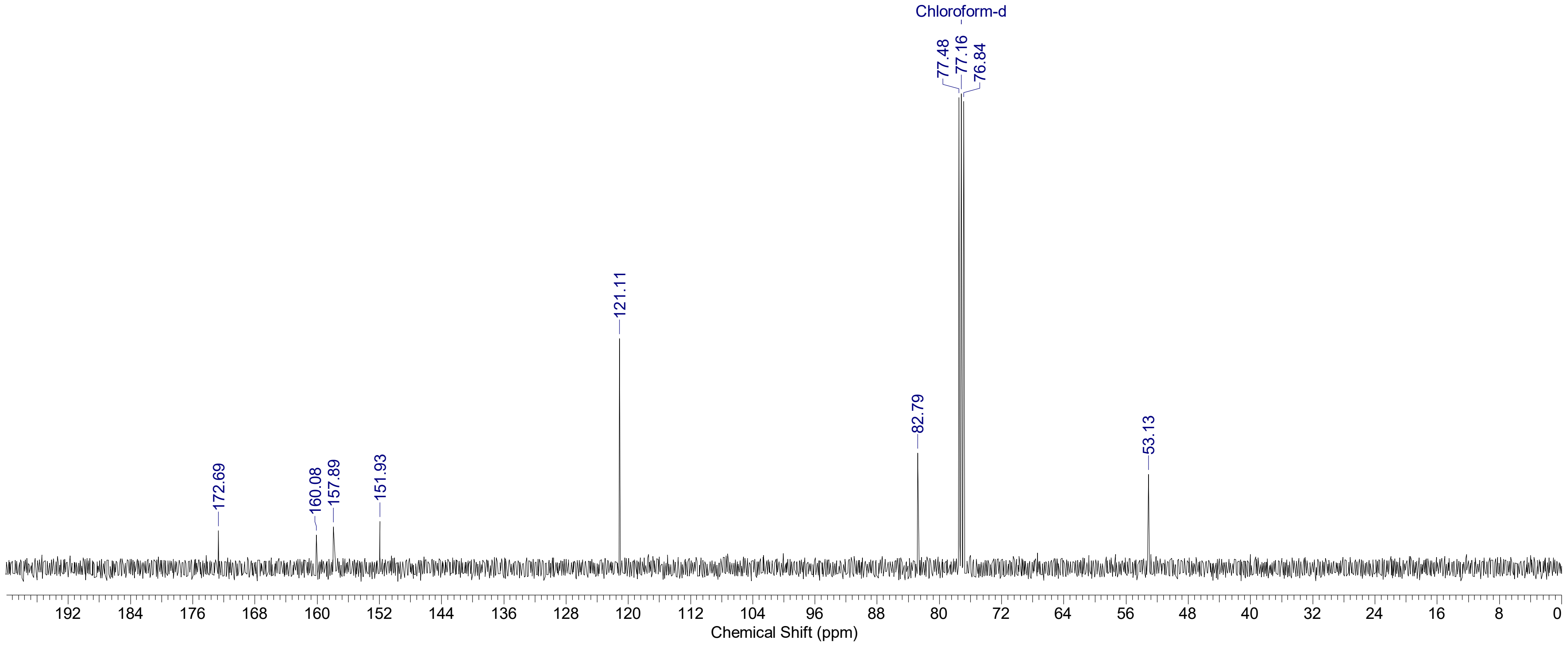

5,5'-(1,4-Phenylenebis(oxy))bis(isoxazole-3-carboxamide) **3c** (^{1}H NMR)

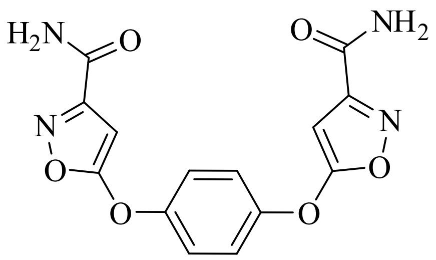

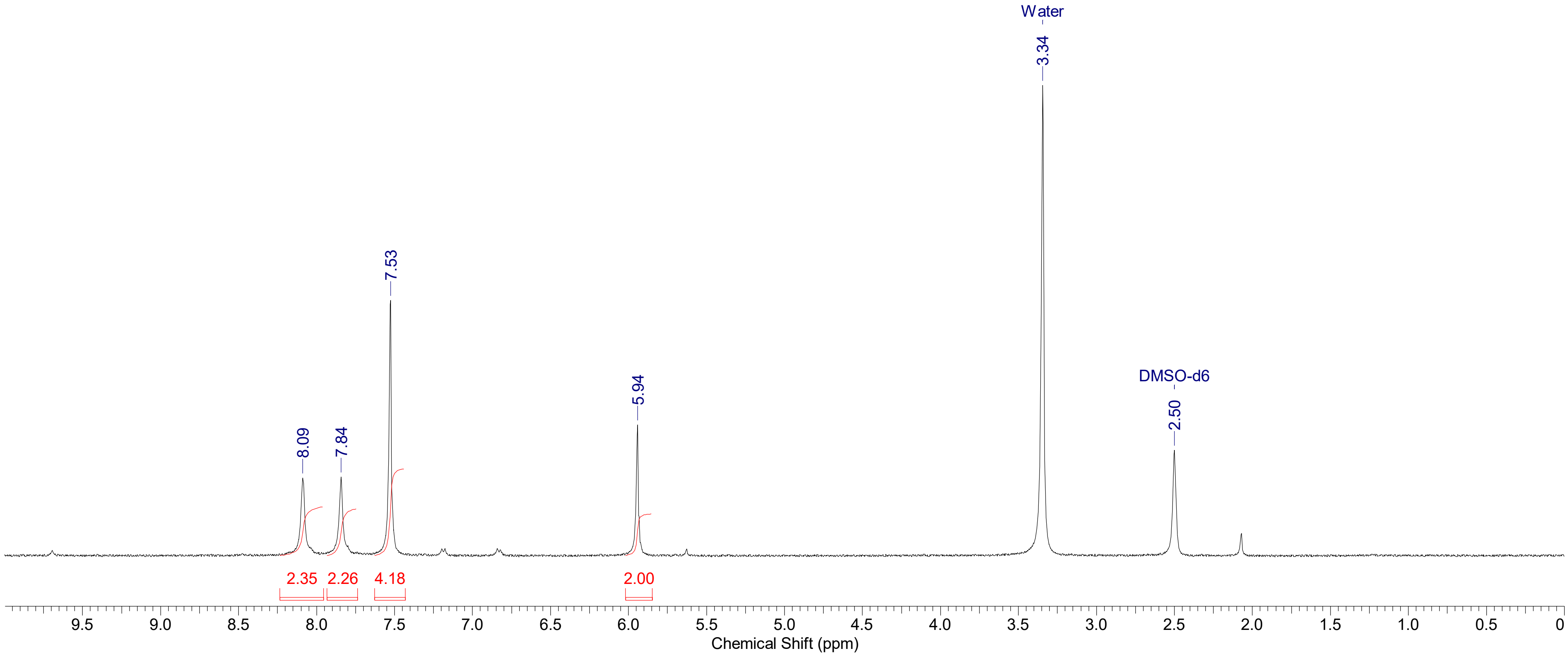

5,5'-(1,4-Phenylenebis(oxy))bis(isoxazole-3-carboxamide) **3c** (^{13}C NMR)

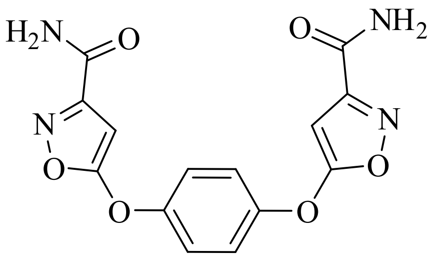

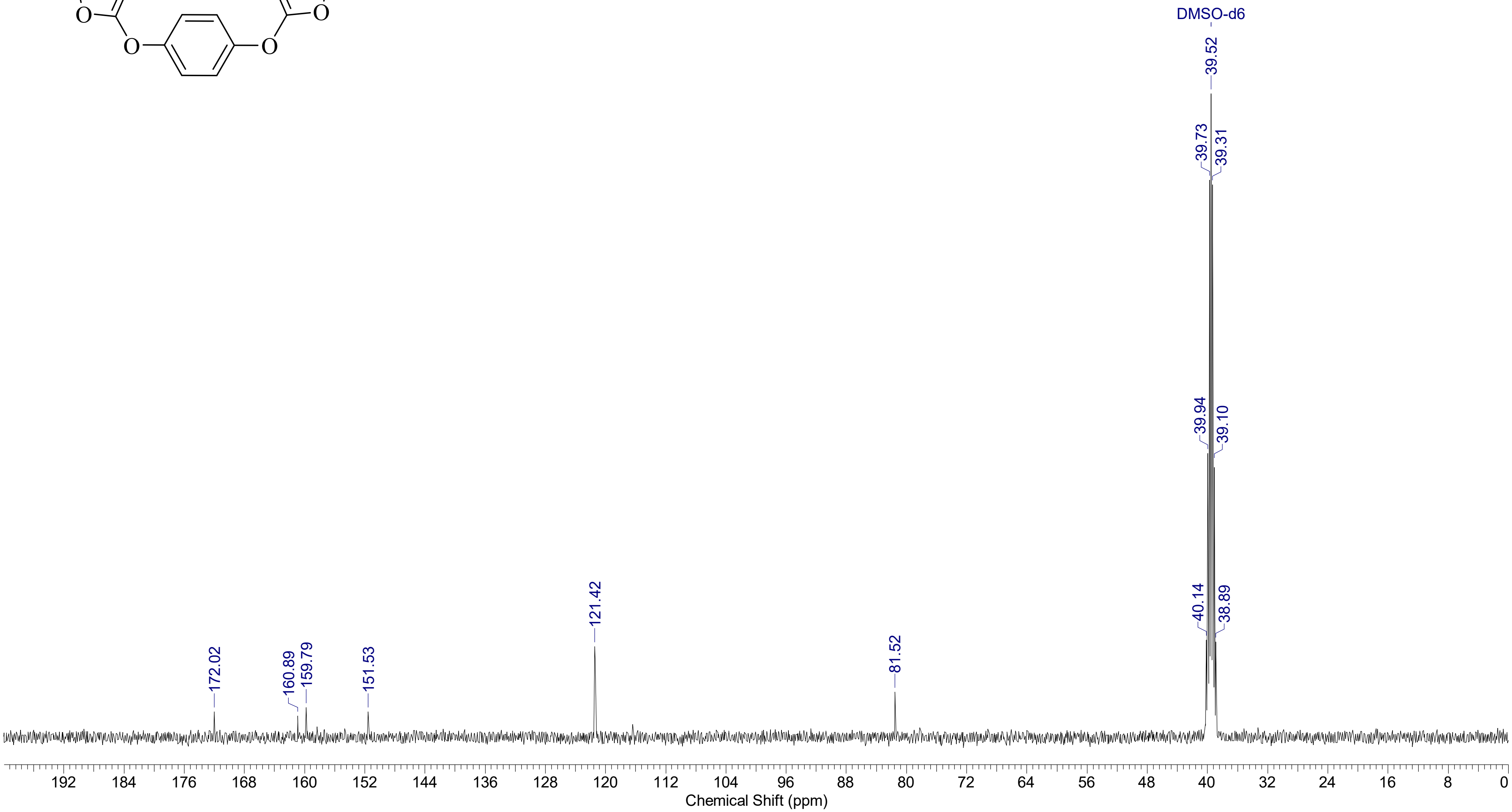

Di-*tert*-butyl 5,5'-(ethane-1,2-diylbis(azanediyl))bis(isoxazole-3-carboxylate) **3d** (^{1}H NMR)

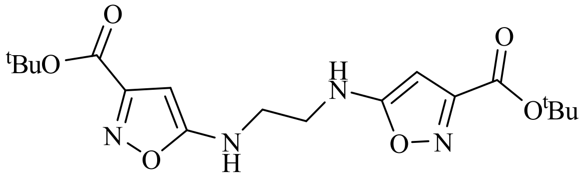

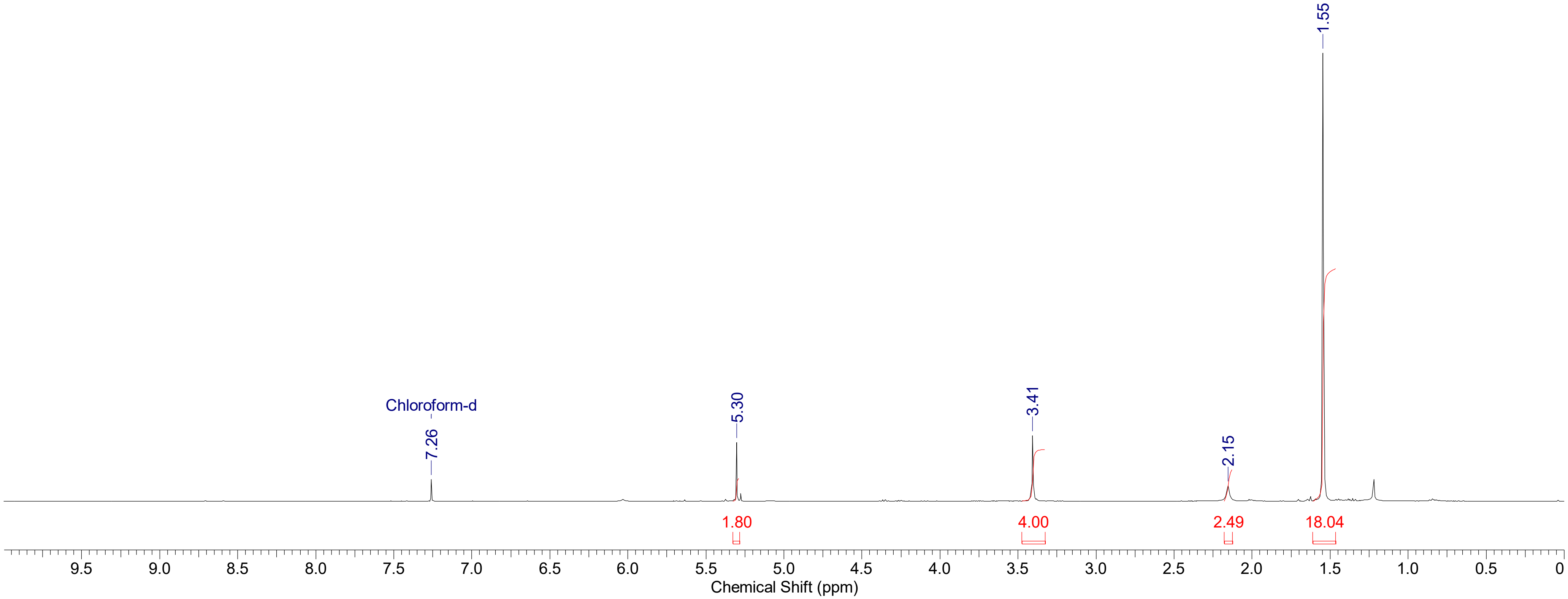

Di-*tert*-butyl 5,5'-(ethane-1,2-diylbis(azanediyl))bis(isoxazole-3-carboxylate) **3d** (^{13}C NMR)

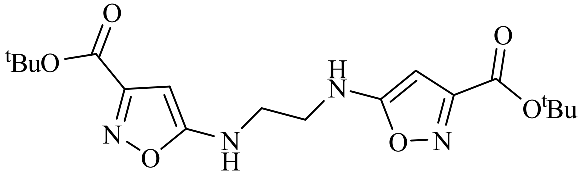

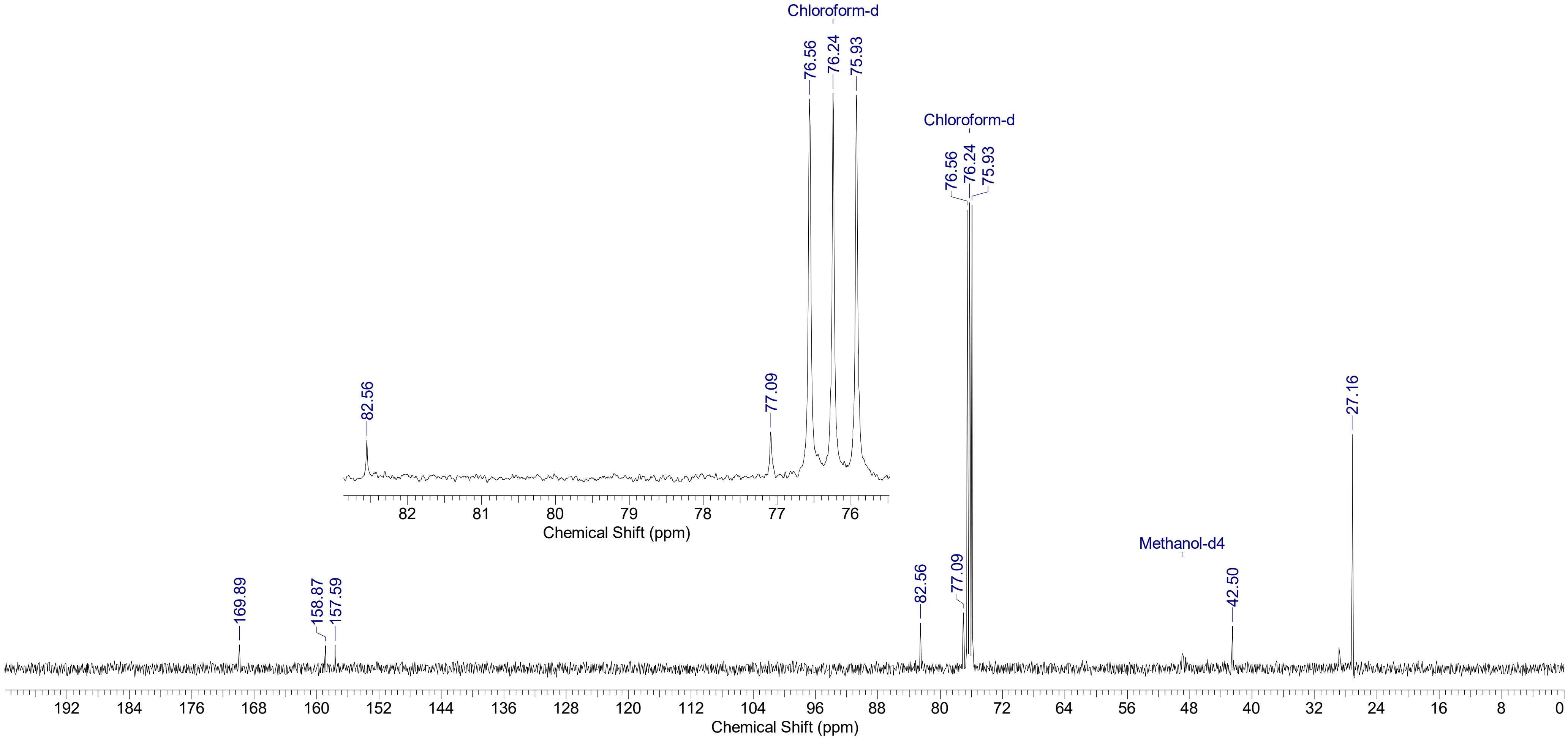

Di-*tert*-butyl 5,5'-(propane-1,3-diylbis(azanediyl))bis(isoxazole-3-carboxylate) **3e** (^{1}H NMR)

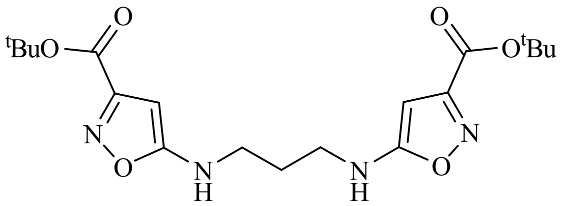

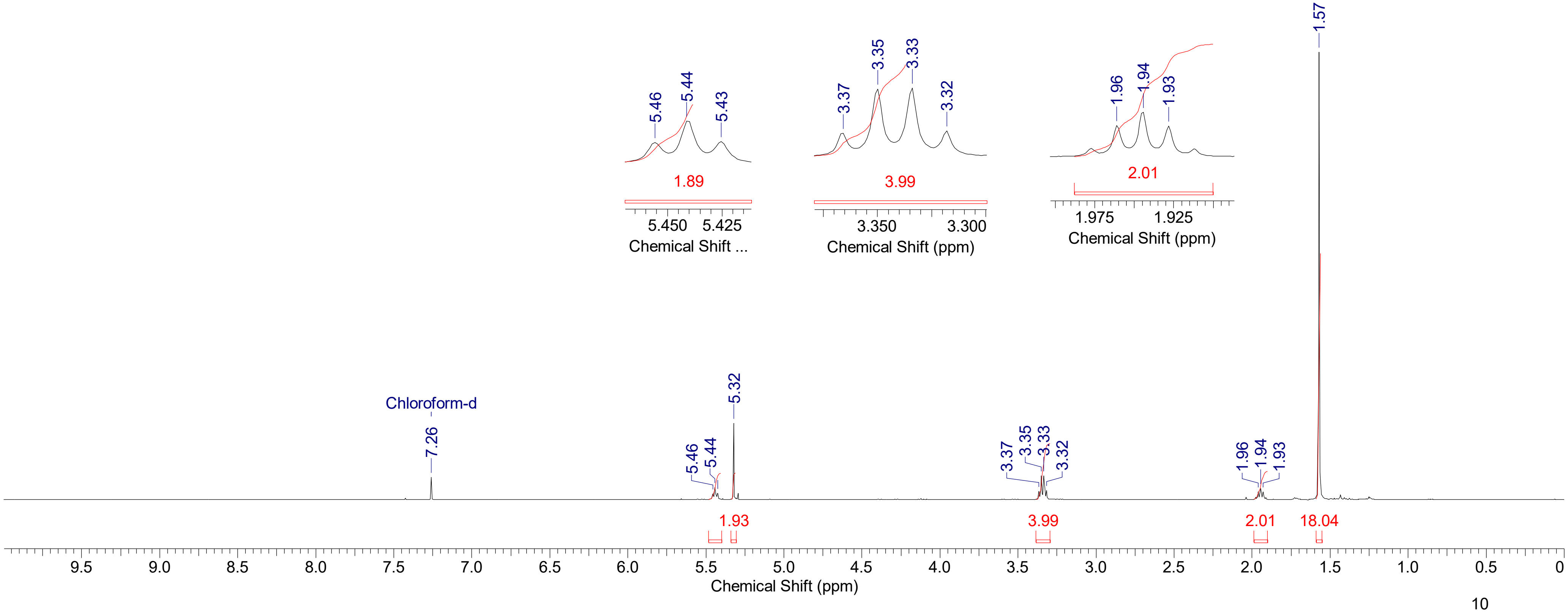

Di-*tert*-butyl 5,5'-(propane-1,3-diylbis(azanediyl))bis(isoxazole-3-carboxylate) **3e** (^{13}C NMR)

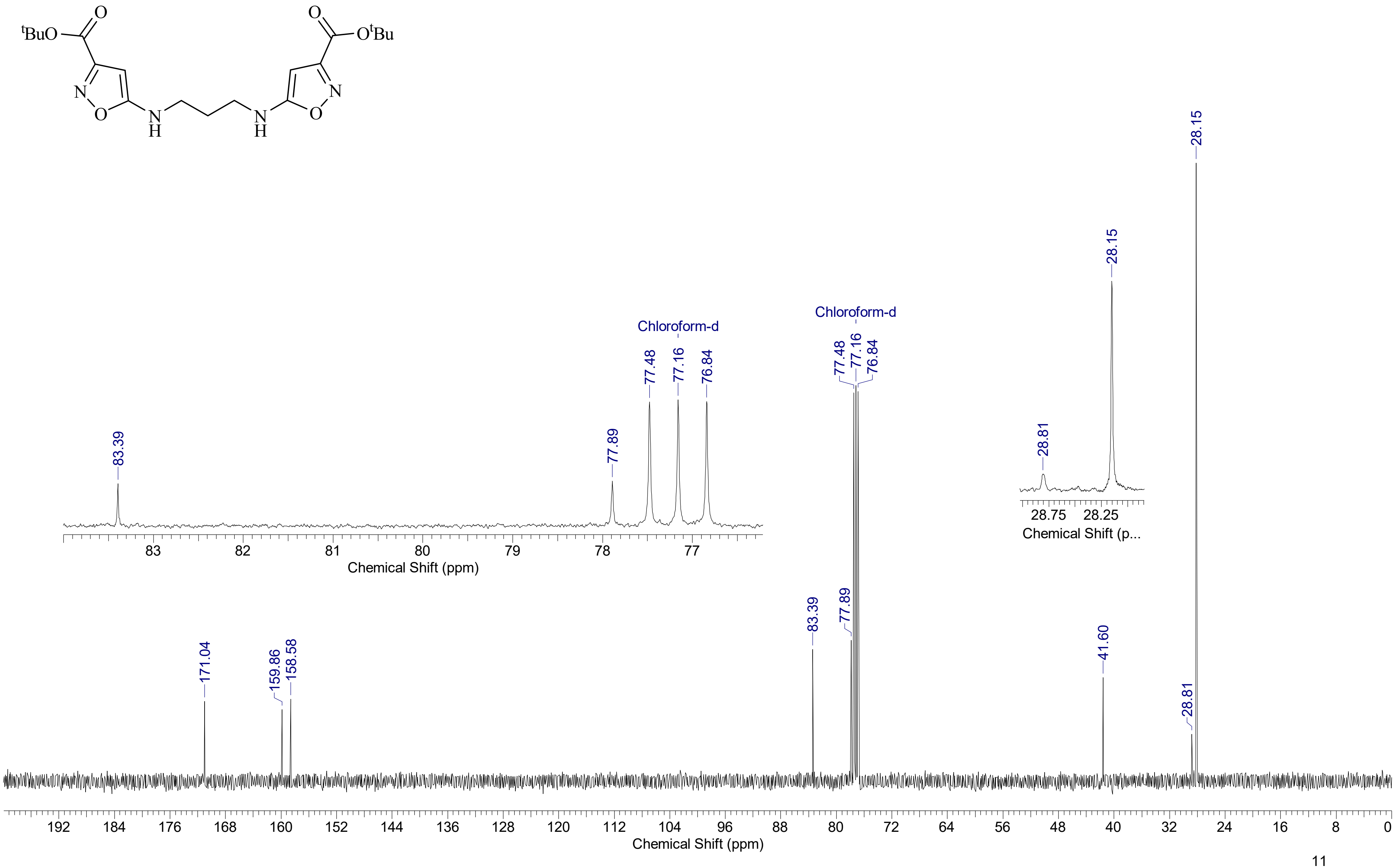

Di-*tert*-butyl 5,5'-(butane-1,4-diylbis(azanediyl))bis(isoxazole-3-carboxylate) **3f** (^{1}H NMR)

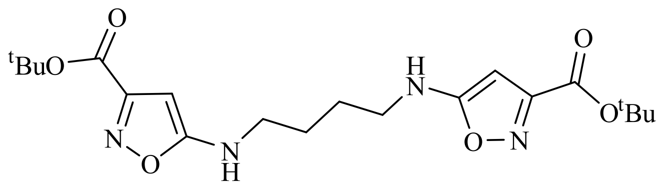

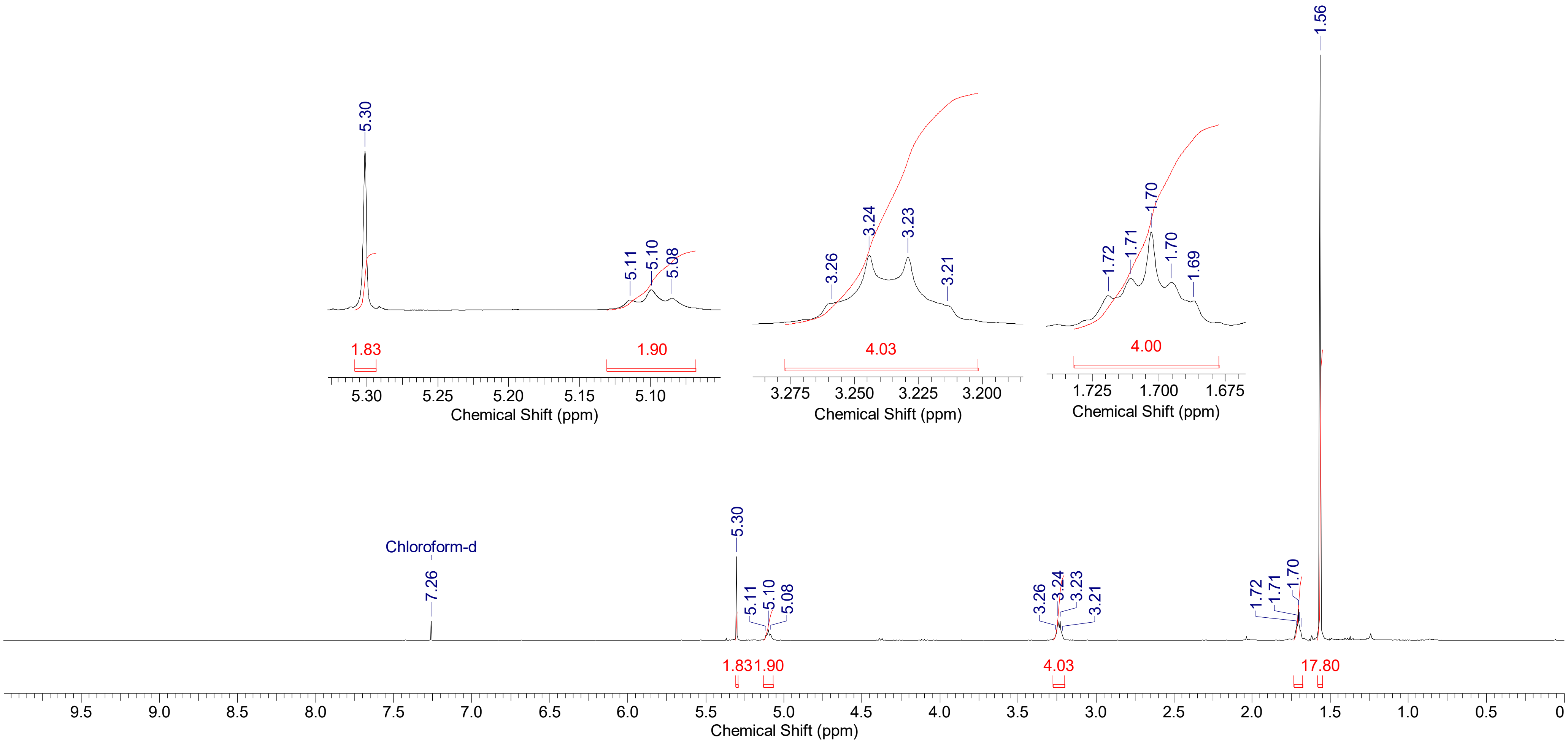

Di-*tert*-butyl 5,5'-(butane-1,4-diylbis(azanediyl))bis(isoxazole-3-carboxylate) **3f** (^{13}C NMR)

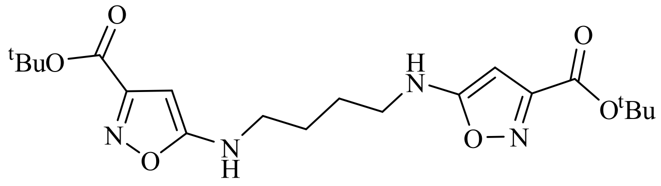

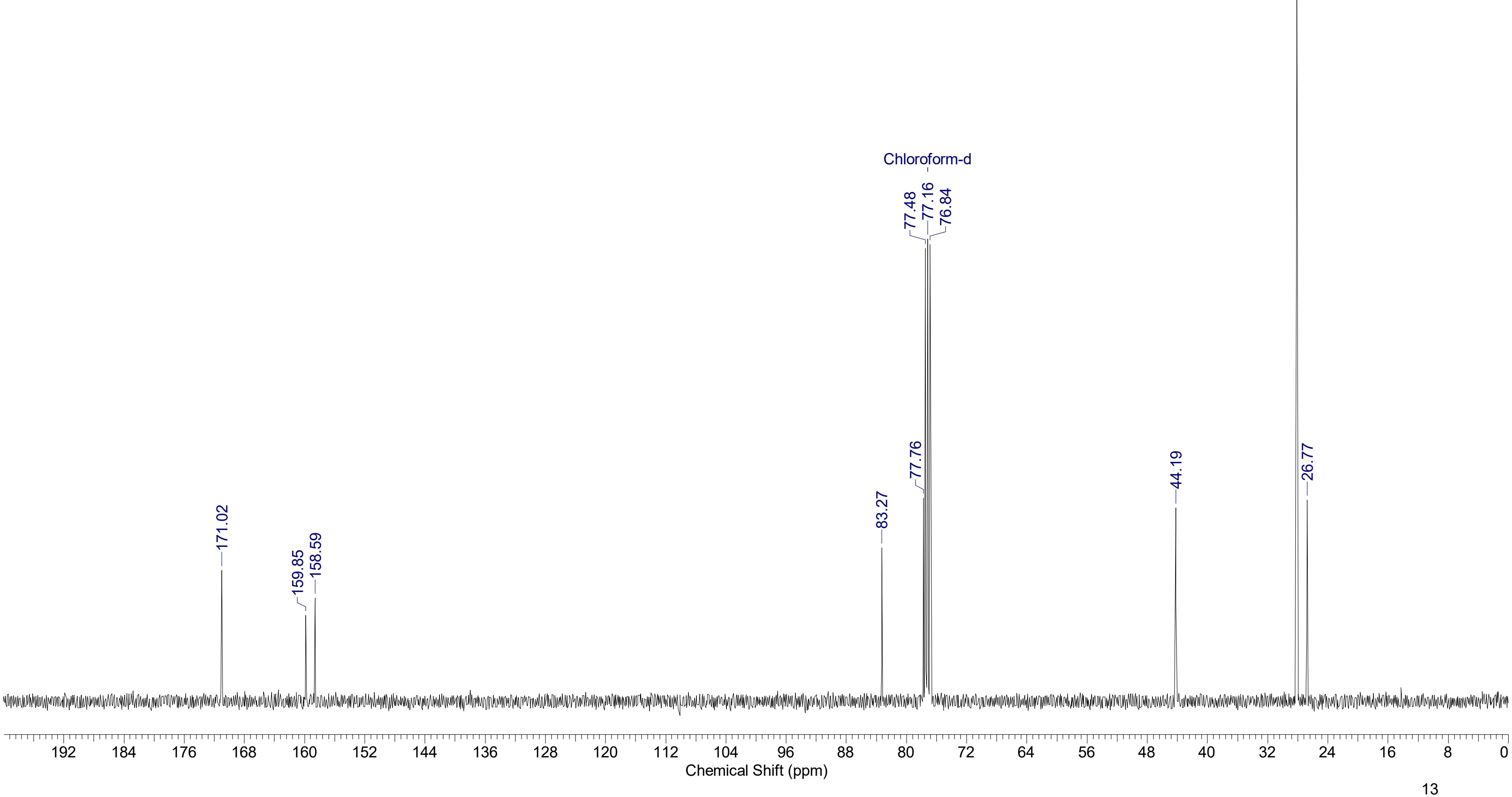

Di-*tert*-butyl 5,5'-(ethane-1,2-diylbis(sulfanediyl))bis(isoxazole-3-carboxylate) **3g** (^{1}H NMR)

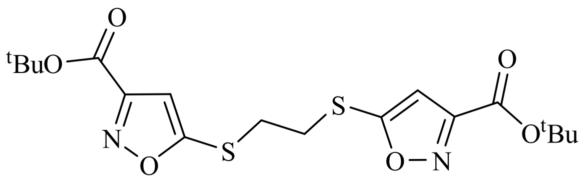

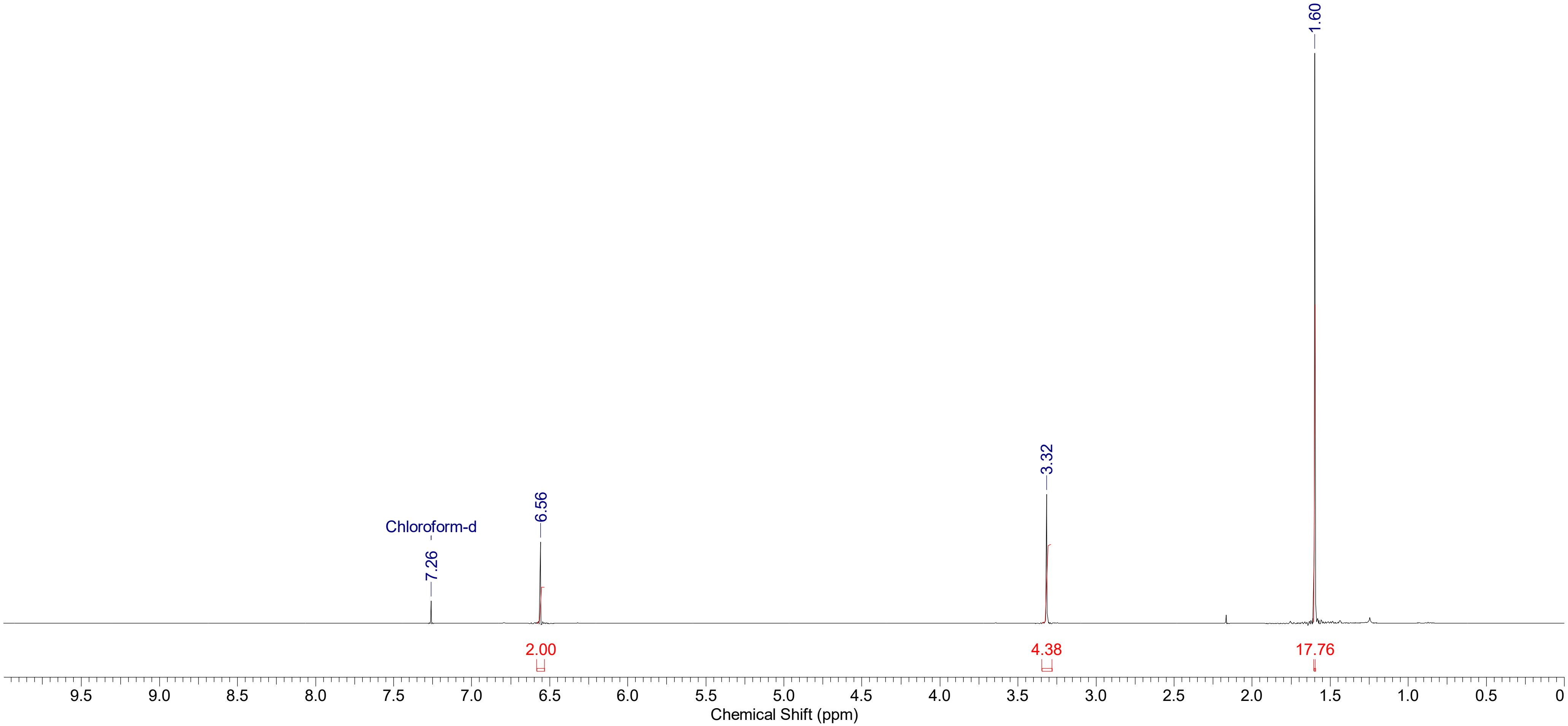

Di-*tert*-butyl 5,5'-(ethane-1,2-diylbis(sulfanediyl))bis(isoxazole-3-carboxylate) **3g** (^{13}C NMR)

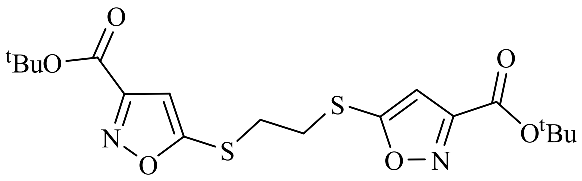

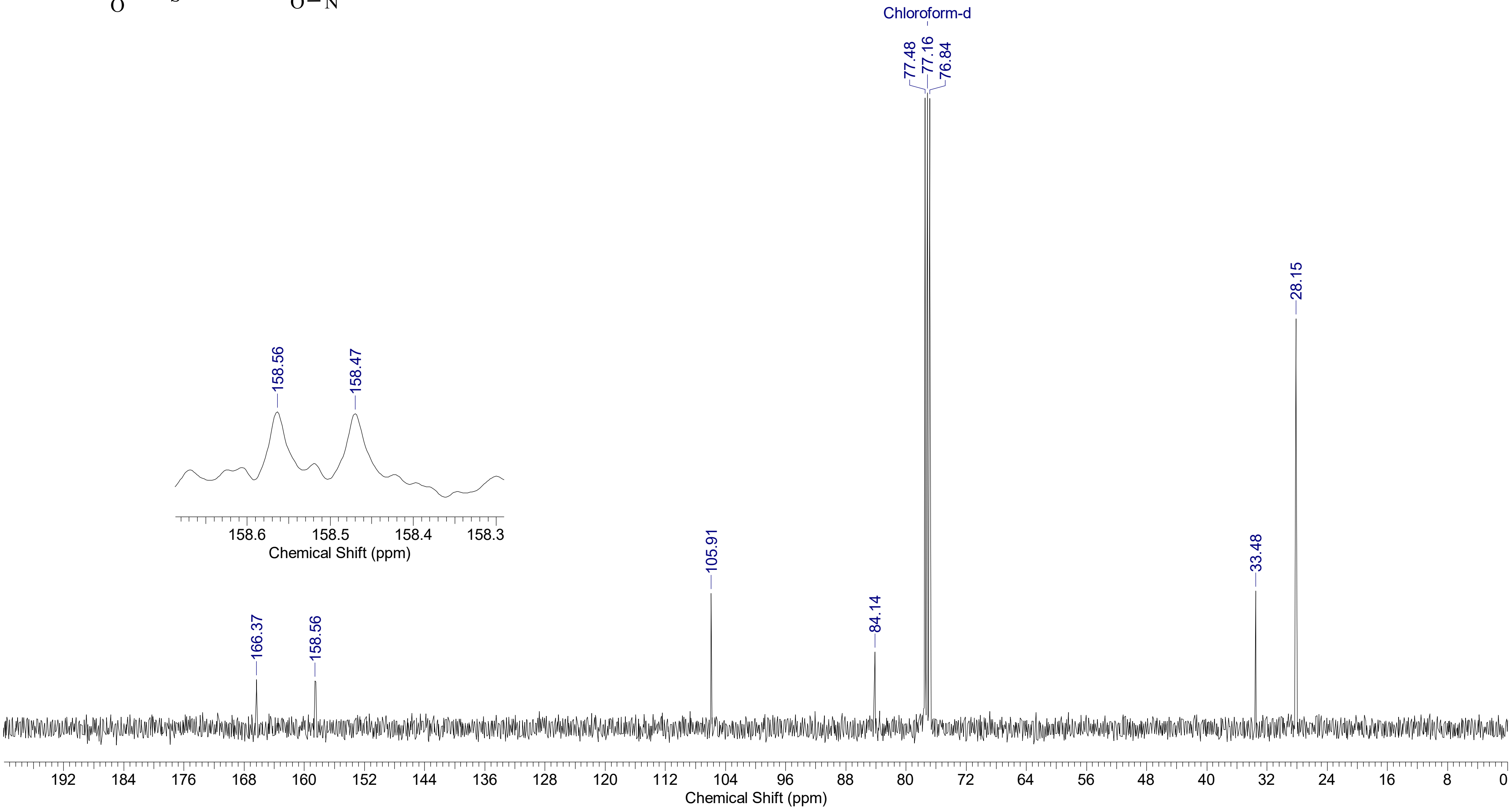

Di-*tert*-butyl 5,5'-(propane-1,3-diylbis(sulfanediyl))bis(isoxazole-3-carboxylate) **3h** (^{1}H NMR)

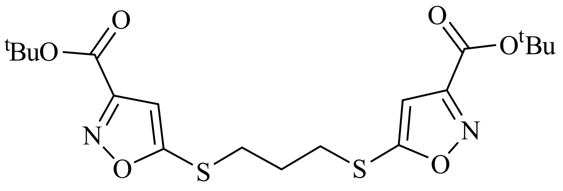

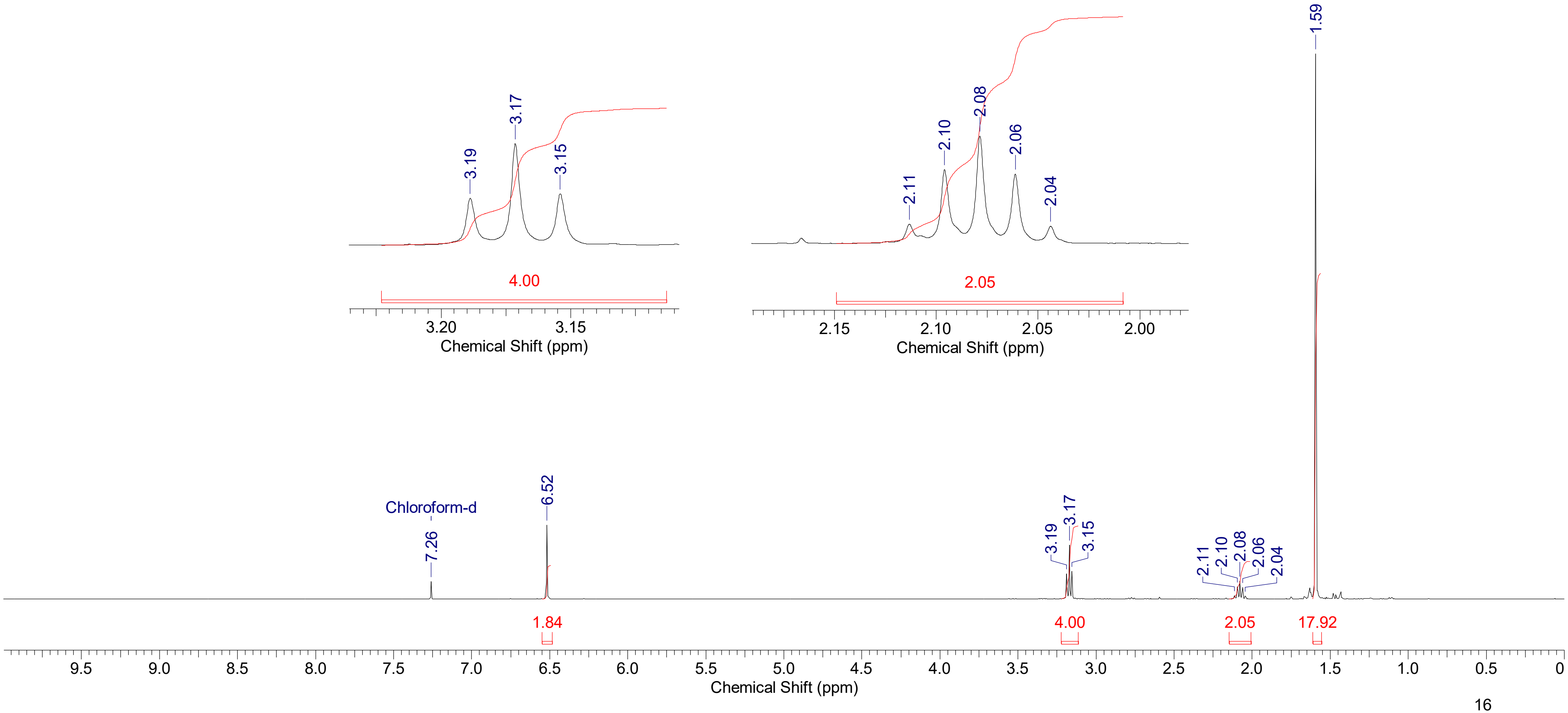

Di-*tert*-butyl 5,5'-(propane-1,3-diylbis(sulfanediyl))bis(isoxazole-3-carboxylate) **3h** (^{13}C NMR)

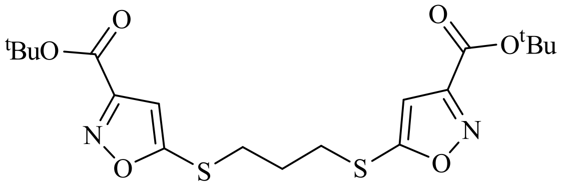

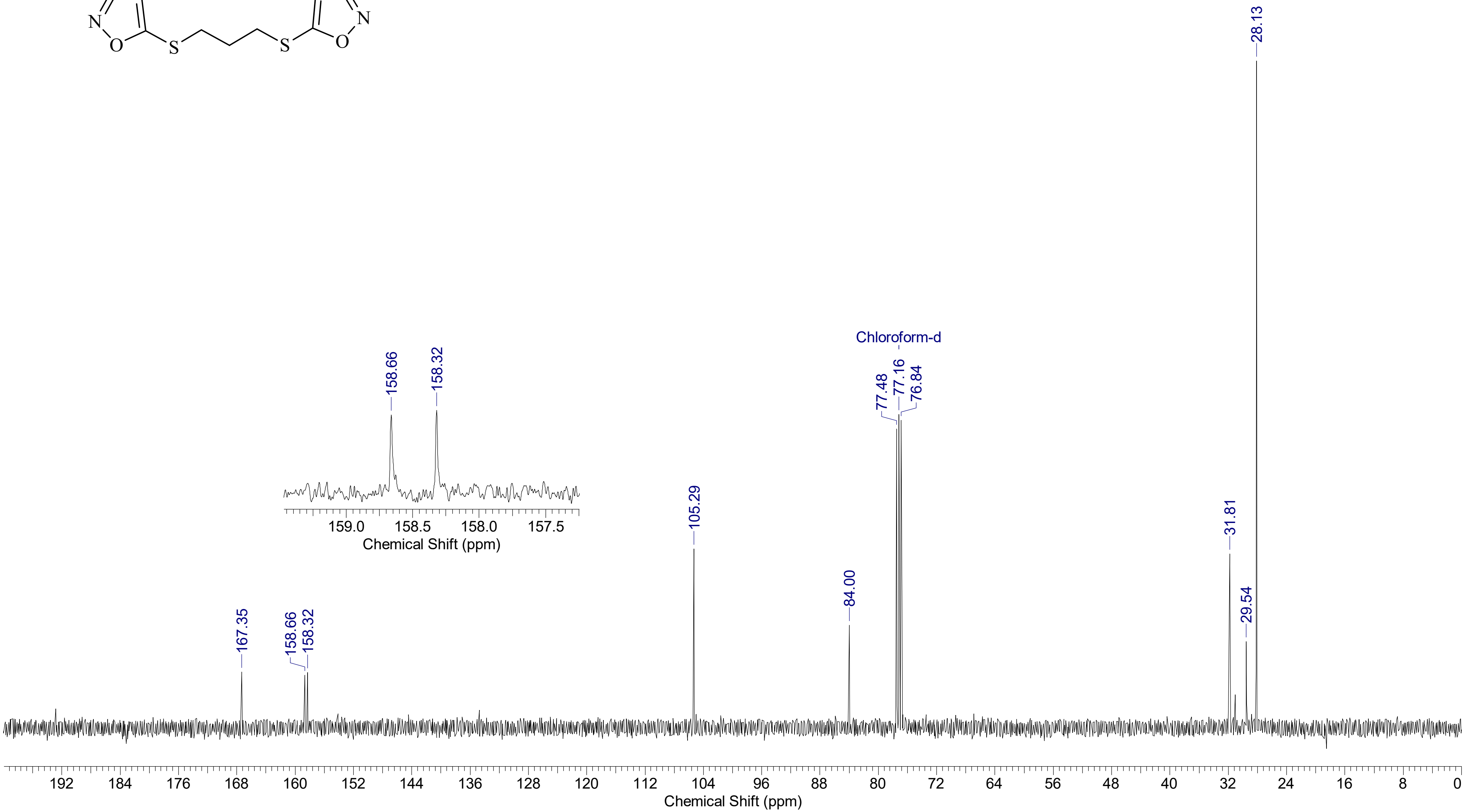

Diisopropyl 5,5'-(ethane-1,2-diylbis(sulfanediyl))bis(isoxazole-3-carboxylate) **3i** (^{1}H NMR)

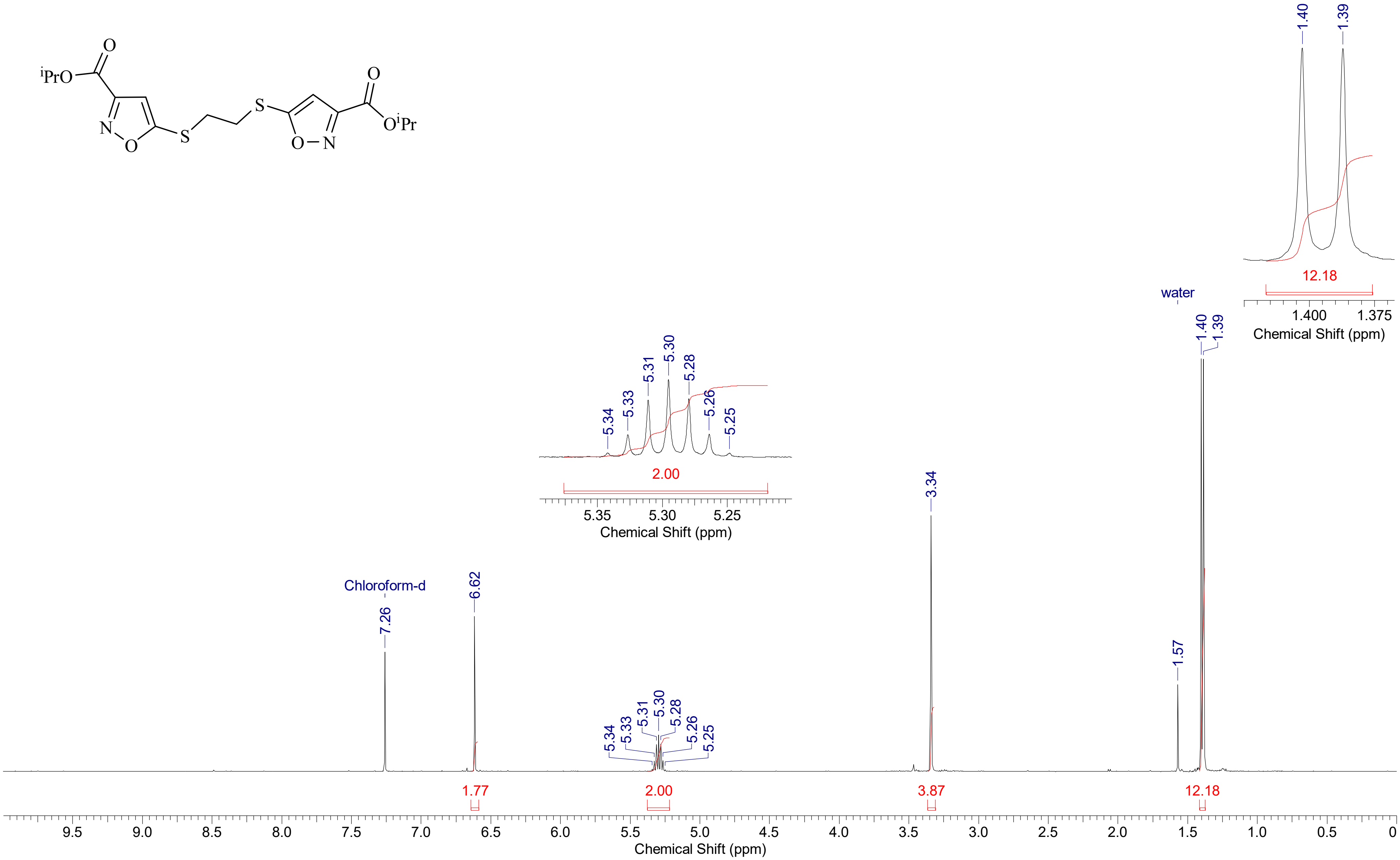

Diisopropyl 5,5'-(ethane-1,2-diylbis(sulfanediyl))bis(isoxazole-3-carboxylate) **3i** (^{13}C NMR)

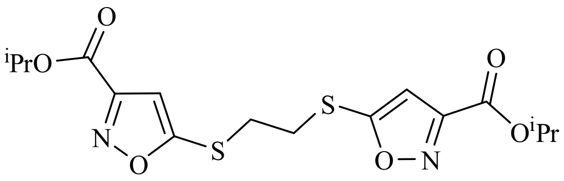

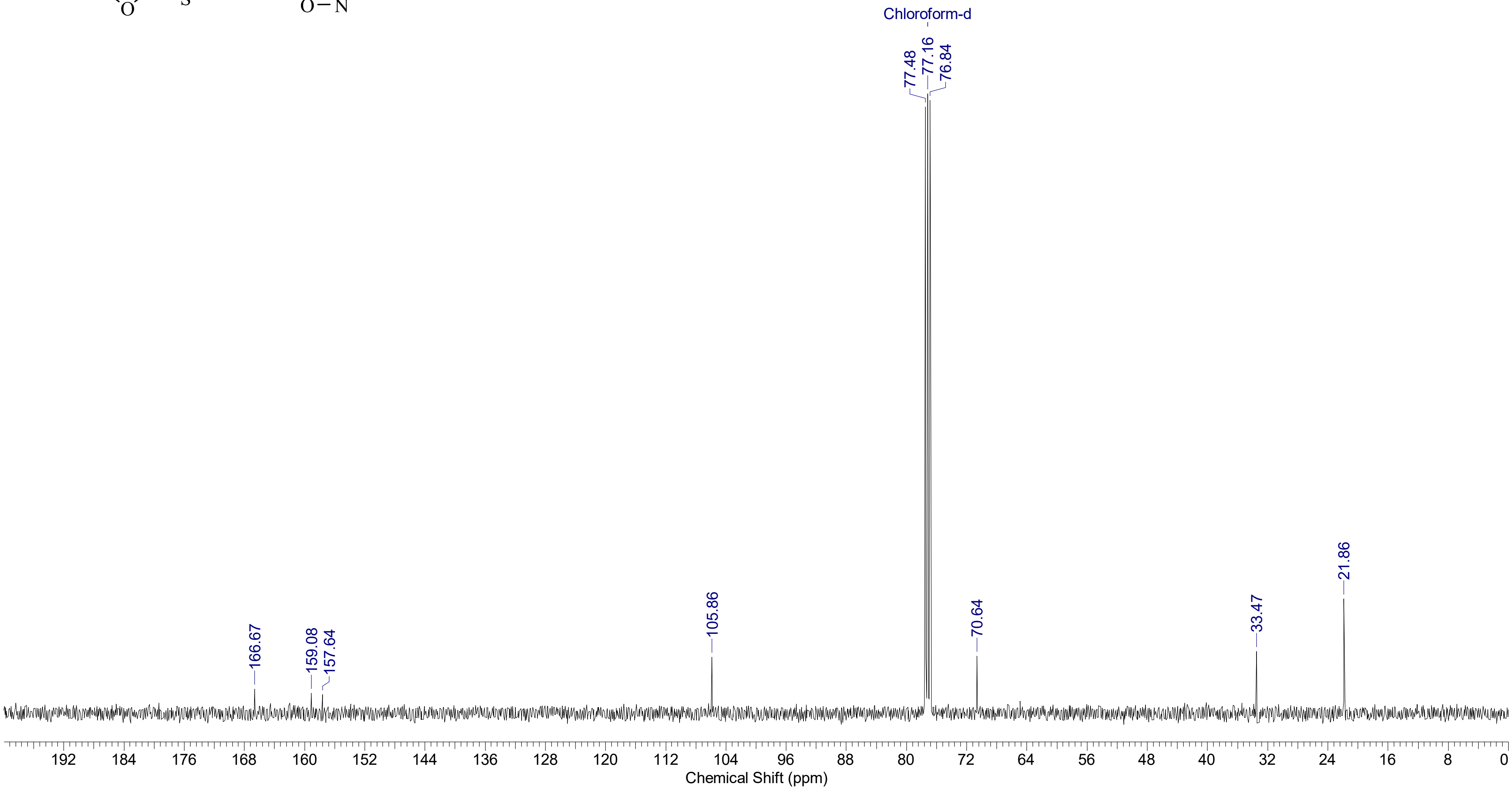

Dimethyl 5,5'-(ethane-1,2-diylbis(sulfanediyl))bis(isoxazole-3-carboxylate) **3j** (^{1}H NMR)

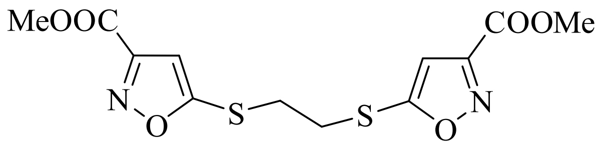

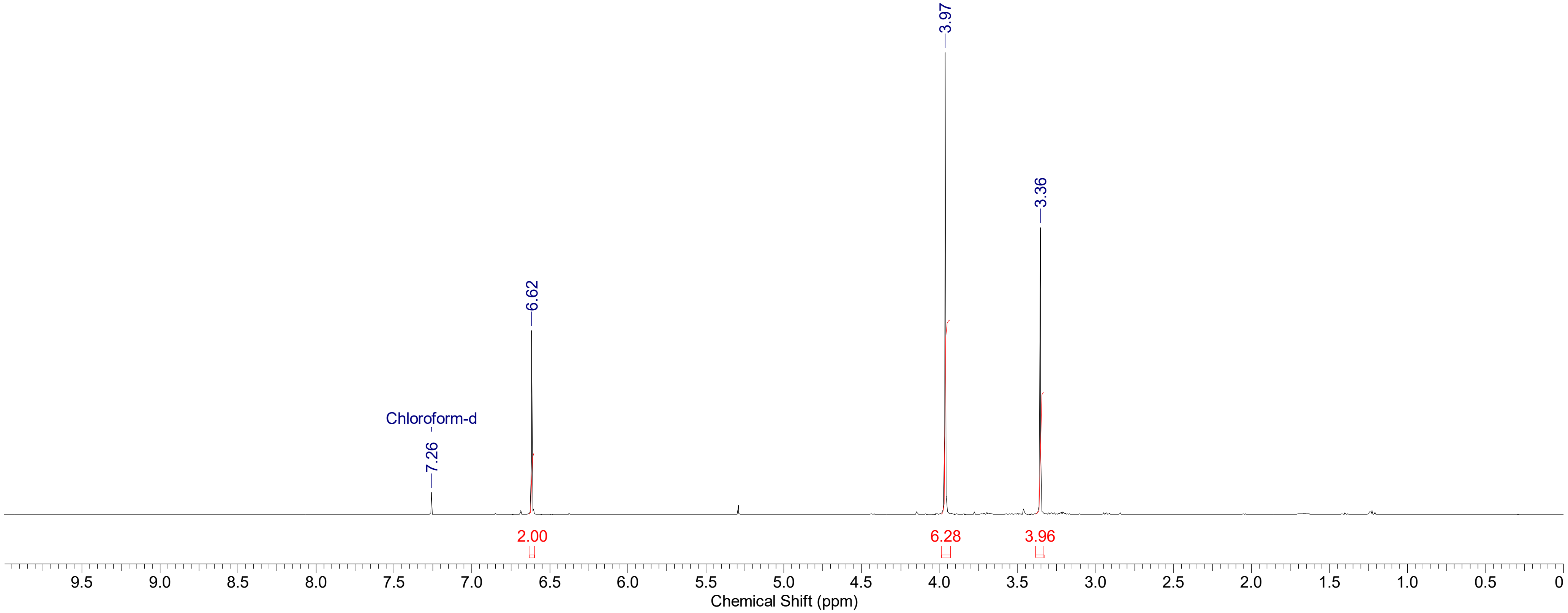

Dimethyl 5,5'-(ethane-1,2-diylbis(sulfanediyl))bis(isoxazole-3-carboxylate) **3j** (^{13}C NMR)

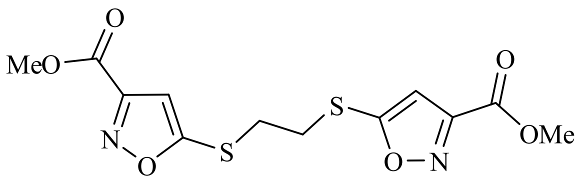

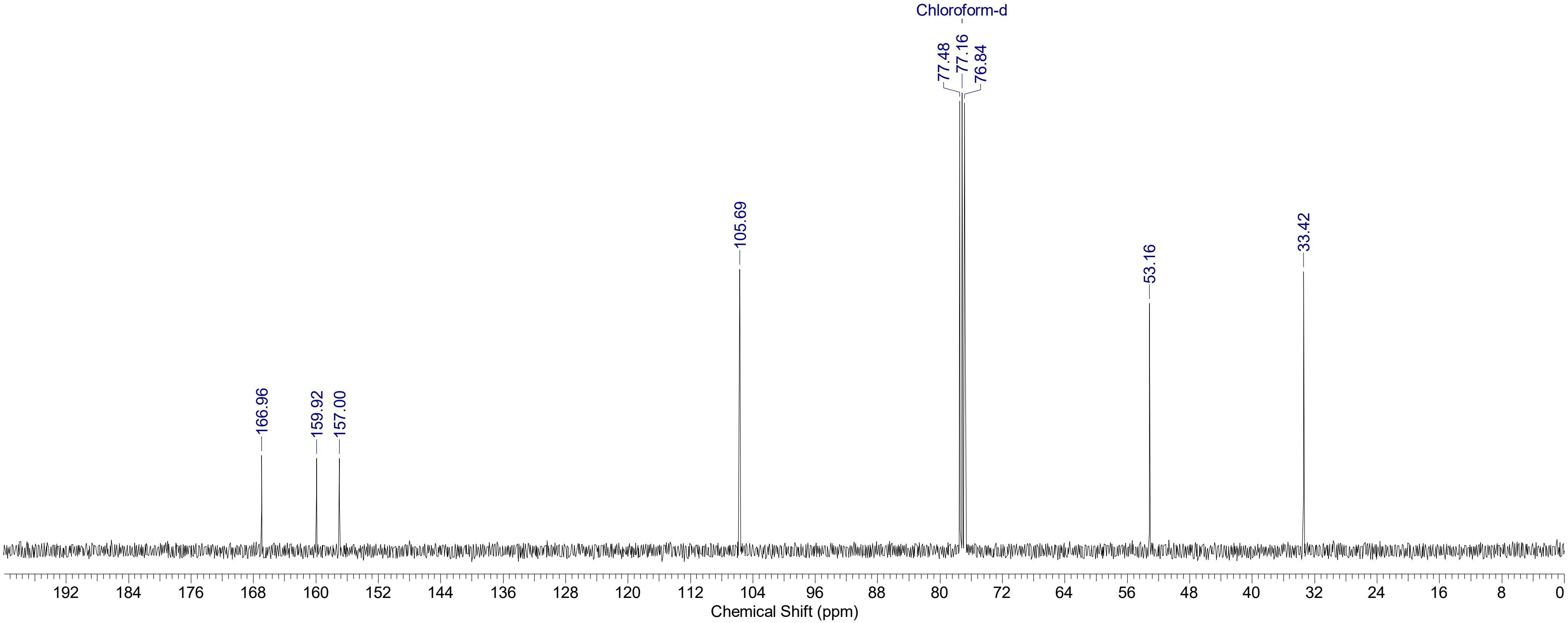

S2. Electrophysiological Evaluation

The AMPA receptor modulator activity of the compounds was evaluated in electrophysiological experiments in vitro using a patch clamp technique with the local fixation of potential, as described earlier [1,2]. In brief, freshly isolated single Purkinje neurons from the cerebellum of 12–15-day-old Wistar rats were used as a test system. Transmembrane currents were induced by the activation of the AMPA receptors with a solution of their partial agonist kainic acid using a fast superfusion of solutions, wherein 30 μL of the agonist buffer (the agonist concentration varied in the range of 10^{-6}–10^{-4} M) was added to the constant flow of the neuron-washing buffer. The applications for the control and for each concentration of a compound were performed in triplicate. The transmembrane currents for the individual neurons were recorded using 2.5–5.5 MΩ borosilicate microelectrodes in a whole-cell configuration with an EPC-9 device (HEKA, Germany). The data were processed using the Pulsfit program (HEKA, Germany). Cyclothiazide (CTZ), a well-known positive allosteric modulator of AMPA receptors, was used as a reference ligand.

S3. Molecular Modeling

The modeling of the ligand interactions with the dimeric ligand-binding domain of the GluA2 AMPA was performed as described earlier [2,3]. Specifically, the protein structure was obtained from the Protein Data Bank (PDB: 4FAT) [4]. Upon removal of the ions and small molecules (except for the two receptor-bound glutamate agonist molecules), the protein was allowed to relax during the molecular dynamics simulation for 100 ns (see below for the simulation protocol). The most frequently occurring structure was identified by the clustering of the frames in the stable part of the trajectory (40–100 ns). The ligand structures were converted to 3D and preoptimized in the MMFF94 force field using Avogadro 1.2.0 software (Avogadro Chemistry, https://avogadro.cc/ accessed on 1 September 2023) [5], and then the protein and ligand structures were prepared for molecular docking using AutoDock Tools 1.5.7 (The Scripps Research Institute, La Jolla, CA, USA, https://ccsb.scripps.edu/mgltools/ accessed on 1 September 2023) [6]. The molecular docking to the positive allosteric modulator binding site was performed with AutoDock Vina 1.1.2 software (The Scripps Research Institute, La Jolla, CA, USA, https://vina.scripps.edu/ accessed on 1 September 2023) [7] (grid box size 22 Å × 29 Å × 40 Å, exhaustiveness = 16). The pose with the best scoring function value and ligand position was selected, and the complex model was built using the USCF Chimera 1.15 software (University of California San Francisco, San Francisco, USA, https://www.cgl.ucsf.edu/chimera/ accessed on 1 September 2023) [8].

The molecular dynamics simulations were performed using the CHARMM36/CGenFF 4.6 force field [9,10] on the GROMACS 2023.0 software (GROMACS development team, https://www.gromacs.org/ accessed on 1 September 2023) [11]. The initial models of the systems were built using the Ligand Reader & Modeler and Solution Builder modules of the CHARMM-GUI web service (https://charmm-gui.org/ accessed on 01 September 2023) [12,13]. The protein molecule was inserted into a rectangular box of water in the TIP3P model; the distance from the protein to the box border was no less than 10 Å. Individual, randomly selected water molecules were replaced with potassium and chlorine ions to ensure the electrical neutrality of the system and the total concentration of KCl of approximately 0.15 M. For each system, the molecular mechanics minimization (up to 5000 steps) was performed on the CPU, followed by equilibration for 125 ps at the temperature of 300 K and a constant volume using the v-rescale thermostat on the NVIDIA GeForce RTX 3080Ti GPU. The production simulation was performed on the GPU at the constant pressure of 1 bar and the temperature of 300 K, using the v-rescale thermostat and the Parrinello–Rahman barostat. The hydrogen atom movements were constrained using the LINCS algorithm. For the analysis and visualization of the results, the CPPTRAJ 6.4.4 software (Daniel R. Roe, Amber development team, http://ambermd.org/ accessed on 1 September 2023) [14] in the AmberTools 22 package [15] and UCSF Chimera were used. The binding free energies were estimated over the stable portion of the trajectories (last 20 ns, 101 frames at 200 ps interval) using the MM/GBSA approach implemented using the gmx_MMPBSA 1.6.1 software (gmx_MMPBSA development team, https://valdes-tresanco-ms.github.io/gmx_MMPBSA/dev/ accessed on 1 September 2023) [16,17]. The internal dielectric constant ε = 4, a salt concentration of 0.15 M, and the interaction entropy model for the conformation entropy contribution were used. The resulting energy values are listed in Table S1.

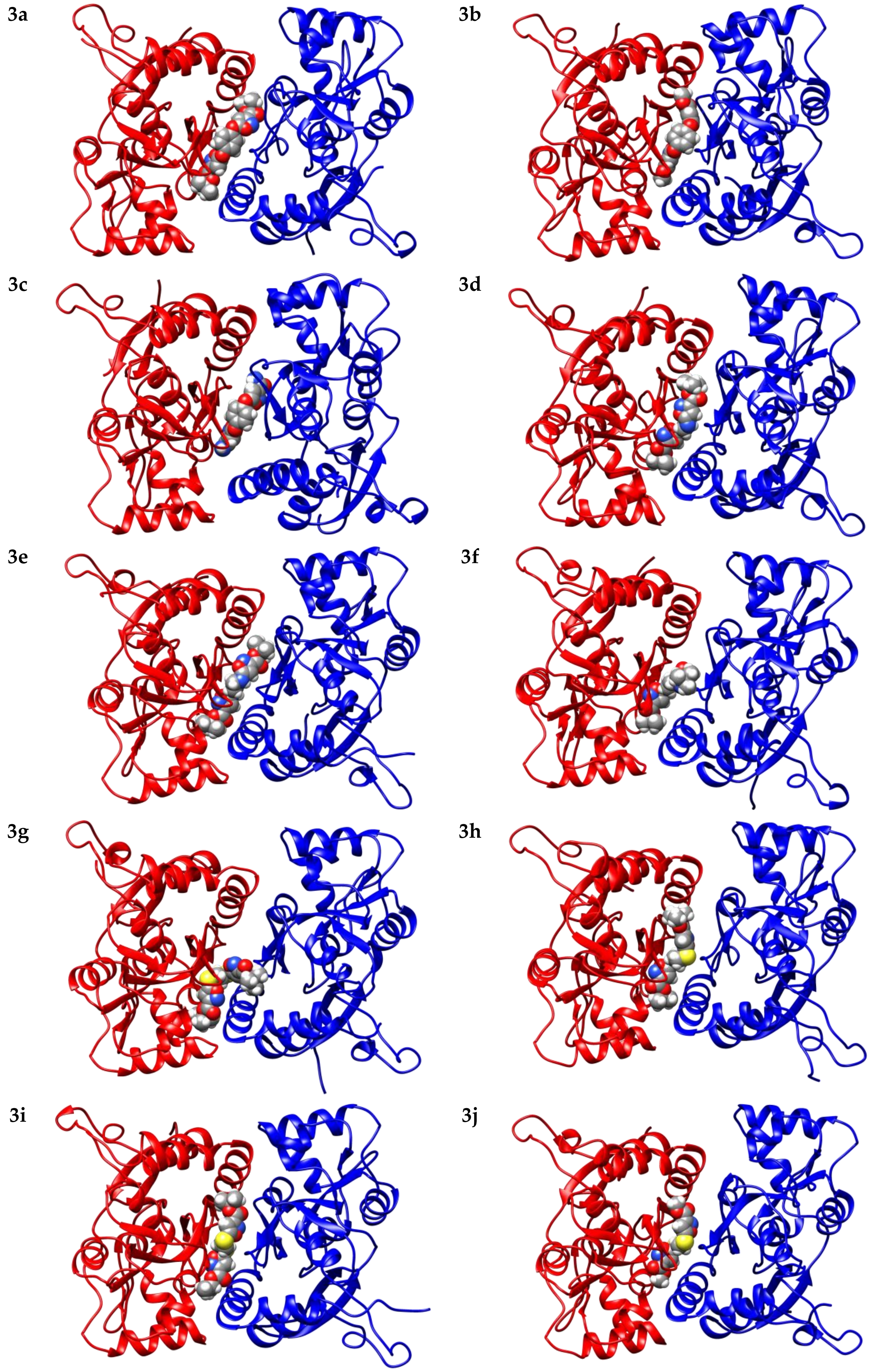

Figure S1. Binding modes of compounds **3a–j** in the PAM binding site refined using molecular dynamics simulations (100 ns). For each compound, a general view of the dimeric ligand binding domain of AMPA receptor (GluA2) and the ligand position are shown.

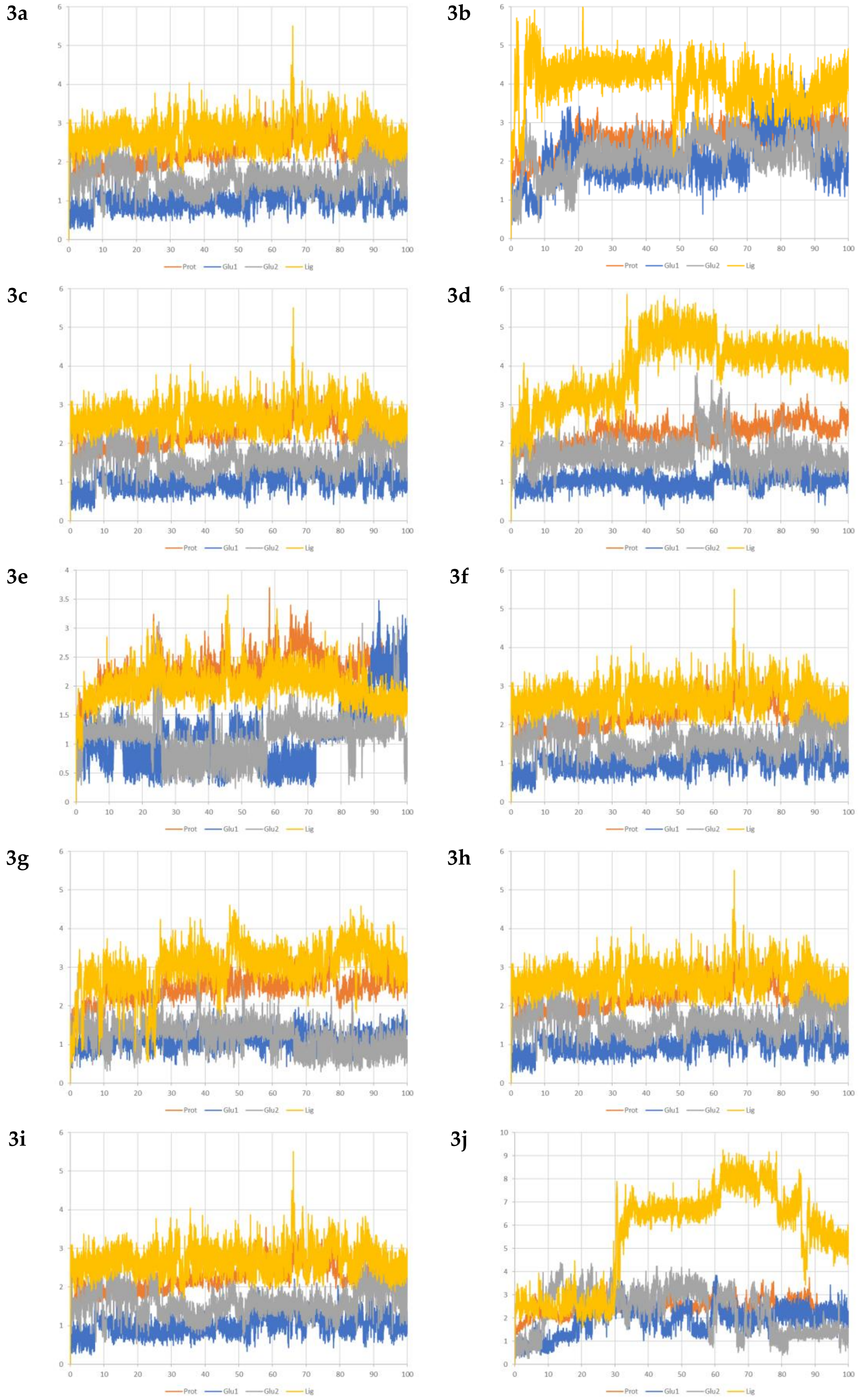

Figure S2. RMSD of the protein, glutamate, and ligand heavy atoms for compounds **3a–j** during molecular dynamics simulations of the modulator complex with the dimeric ligand binding domain of the GluA2 AMPA receptor (RMSD, Å; Time, ns).

Table S1. Modulator activity profiles, MM/GBSA binding energies, and geometrical parameters of subunit arrangement for compounds **3a–j** during molecular dynamics simulations of the modulator complex with the dimeric ligand binding domain of the GluA2 AMPA receptor. (MM/GBSA ΔG, kcal/mol; angleAB, degrees; distances, Å).

Compound	Activity	MMGBSA	angleAB	heightFaceAB	distFace	shiftFaceAB	heightS_AB	shiftSL_AB	heightL_AB	shiftLS_AB
**3a**	Neg	–46.0	8.9	9.7	10.4	3.7	8.8	3.8	9.3	2.6
**3b**	NA	–43.2	4.5	9.2	9.5	2.3	9.6	1.9	9.4	2.6
**3c**	NA	–41.6	17.4	10.4	10.7	2.2	11.9	2.0	11.2	4.6
**3d**	Pos 27	–48.3	4.3	9.5	9.9	2.9	9.8	2.5	9.6	3.0
**3e**	Neg	–40.9	7.8	9.7	10.2	3.0	8.9	3.4	9.3	2.3
**3f**	Neg	–36.8	8.4	9.6	10.0	2.8	8.8	3.2	9.1	2.0
**3g**	Pos 68	–44.7	11.5	10.0	10.5	3.1	8.9	3.9	9.4	2.2
**3h**	Pos 59	–35.3	8.8	9.7	10.2	3.1	8.8	3.6	9.3	2.3
**3i**	Pos 20	–42.3	4.8	9.3	9.6	2.2	8.9	2.9	9.1	2.2
**3j**	Pos 77	–32.1	10.8	9.8	10.1	2.6	8.8	3.2	9.2	1.7

References

1. Lavrov, M.I.; Karlov, D.S.; Voronina, T.A.; Grigoriev, V.V.; Ustyugov, A.A.; Bachurin, S.O.; Palyulin, V.A. Novel positive allosteric modulators of AMPA receptors based on 3,7-diazabicyclo[3.3.1]nonane scaffold. *Mol. Neurobiol.* **2020**, *57*, 191–199, doi:10.1007/s12035-019-01768-6.
2. Vasilenko, D.A.; Sadovnikov, K.S.; Sedenkova, K.N.; Karlov, D.S.; Radchenko, E.V.; Grishin, Y.K.; Rybakov, V.B.; Kuznetsova, T.S.; Zamoyski, V.L.; Grigoriev, V.V.; et al. A facile approach to bis(isoxazoles), promising ligands of the AMPA receptor. *Molecules* **2021**, *26*, 6411, doi:10.3390/molecules26216411.
3. Lavrov, M.I.; Veremeeva, P.N.; Golubeva, E.A.; Radchenko, E.V.; Zamoyski, V.L.; Grigoriev, V.V.; Palyulin, V.A. Positive and negative AMPA receptor modulators based on tricyclic bispidine derivative: Minor structural change inverts the type of activity. *Mendeleev Commun.* **2022**, *32*, 360–363, doi:10.1016/j.mencom.2022.05.023.
4. Harms, J.E.; Benveniste, M.; Maclean, J.K.F.; Partin, K.M.; Jamieson, C. Functional analysis of a novel positive allosteric modulator of AMPA receptors derived from a structure-based drug design strategy. *Neuropharmacology* **2013**, *64*, 45–52, doi:10.1016/j.neuropharm.2012.06.008.
5. Hanwell, M.D.; Curtis, D.E.; Lonie, D.C.; Vandermeersch, T.; Zurek, E.; Hutchison, G.R. Avogadro: an advanced semantic chemical editor, visualization, and analysis platform. *J. Cheminform.* **2012**, *4*, 17, doi:10.1186/1758-2946-4-17.
6. Morris, G.M.; Huey, R.; Lindstrom, W.; Sanner, M.F.; Belew, R.K.; Goodsell, D.S.; Olson, A.J. AutoDock4 and AutoDockTools4: Automated docking with selective receptor flexibility. *J. Comput. Chem.* **2009**, *30*, 2785–2791, doi:10.1002/jcc.21256.
7. Trott, O.; Olson, A.J. AutoDock Vina: improving the speed and accuracy of docking with a new scoring function, efficient optimization, and multithreading. *J. Comput. Chem.* **2010**, *31*, 455–461, doi:10.1002/jcc.21334.
8. Pettersen, E.F.; Goddard, T.D.; Huang, C.C.; Couch, G.S.; Greenblatt, D.M.; Meng, E.C.; Ferrin, T.E. UCSF Chimera – a visualization system for exploratory research and analysis. *J. Comput. Chem.* **2004**, *25*, 1605–1612, doi:10.1002/jcc.20084.
9. Huang, J.; MacKerell, A.D. CHARMM36 all-atom additive protein force field: validation based on comparison to NMR data. *J. Comput. Chem.* **2013**, *34*, 2135–2145, doi:10.1002/jcc.23354.
10. Vanommeslaeghe, K.; Hatcher, E.; Acharya, C.; Kundu, S.; Zhong, S.; Shim, J.; Darian, E.; Guvench, O.; Lopes, P.; Vorobyov, I.; et al. CHARMM general force field: A force field for drug-like molecules compatible with the CHARMM all-atom additive biological force fields. *J. Comput. Chem.* **2010**, *31*, 671–690, doi:10.1002/jcc.21367.
11. Abraham, M.J.; Murtola, T.; Schulz, R.; Páll, S.; Smith, J.C.; Hess, B.; Lindahl, E. GROMACS: High performance molecular simulations through multi-level parallelism from laptops to supercomputers. *SoftwareX* **2015**, *1–2*, 19–25, doi:10.1016/j.softx.2015.06.001.
12. Jo, S.; Kim, T.; Iyer, V.G.; Im, W. CHARMM-GUI: a web-based graphical user interface for CHARMM. *J. Comput. Chem.* **2008**, *29*, 1859–1865, doi:10.1002/jcc.20945.
13. Lee, J.; Cheng, X.; Swails, J.M.; Yeom, M.S.; Eastman, P.K.; Lemkul, J.A.; Wei, S.; Buckner, J.; Jeong, J.C.; Qi, Y.; et al. CHARMM-GUI input generator for NAMD, GROMACS, AMBER, OpenMM, and CHARMM/OpenMM simulations using the CHARMM36 additive force field. *J. Chem. Theory Comput.* **2016**, *12*, 405–413, doi:10.1021/acs.jctc.5b00935.
14. Roe, D.R.; Cheatham, T.E. PTRAJ and CPPTRAJ: Software for processing and analysis of molecular dynamics trajectory data. *J. Chem. Theory Comput.* **2013**, *9*, 3084–3095, doi:10.1021/ct400341p.
15. Salomon-Ferrer, R.; Case, D.A.; Walker, R.C. An overview of the Amber biomolecular simulation package. *WIREs Comput. Mol. Sci.* **2013**, *3*, 198–210, doi:10.1002/wcms.1121.
16. Valdés-Tresanco, M.S.; Valdés-Tresanco, M.E.; Valiente, P.A.; Moreno, E. gmx_MMPBSA: A new tool to perform end-state free energy calculations with GROMACS. *J. Chem. Theory Comput.* **2021**, *17*, 6281–6291, doi:10.1021/acs.jctc.1c00645.
17. Miller, B.R.; McGee, T.D.; Swails, J.M.; Homeyer, N.; Gohlke, H.; Roitberg, A.E. MMPBSA.py: An efficient program for end-state free energy calculations. *J. Chem. Theory Comput.* **2012**, *8*, 3314–3321, doi:10.1021/ct300418h.
